# Rutin-Functionalized Selenium Nanoparticles Attenuate Cisplatin-Induced Cardiohepatic Injury in Rats: Modulation of ER Stress-Related Gene Expression and Mitochondrial Apoptotic Markers

**DOI:** 10.3390/ijms27167430

**Published:** 2026-08-19

**Authors:** Nievin Ahmed Mahran, Khaled M. Alam-ELDein, Mohamed A. Ali, Khalid M. Alsyaad, Rabab Mohamed Aljarari, Reem S. Alazragi, Maha Alsunbul, Salma M. Selim, Ossama M. Sayed, Mohamed H. A. Gadelmawla, Samah Shehata Mohammed

**Affiliations:** 1 Department of Biochemistry, Faculty of Biotechnology, Sinai University, Kantara Branch, Ismailia 41636, Egypt; 2 School of Biotechnology, Badr University in Cairo, Cairo 11829, Egypt; 3 Department of Biology, College of Science, King Khalid University, Abha 61413, Saudi Arabia; 4 Department of Biological Sciences, College of Science, University of Jeddah, Jeddah 21589, Saudi Arabia; 5 Department of Pharmaceutical Sciences, College of Pharmacy, Princess Nourah bint Abdulrahman University, P.O. Box 84428, Riyadh 11671, Saudi Arabia; 6 Department of Pharmacology & Toxicology, Faculty of Pharmacy, Sinai University, Kantara Branch, Ismailia 41636, Egypt; 7 Department of Pharmaceutics, Faculty of Pharmacy, Sinai University, Kantara Branch, Ismailia 41612, Egypt; 8 Life Sciences Department, Faculty of Biotechnology, Sinai University, Kantara Branch, Ismailia 41636, Egypt; 9 Biochemistry Department, Faculty of Pharmacy, Fayoum University, Fayoum 63514, Egypt

**Keywords:** cisplatin-induced organ toxicity, rutin-mediated selenium nanoparticles, hepato-cardiotoxicity, KEAP-1/Nrf2 signaling pathway, PERK/ATF4/CHOP signaling pathway

## Abstract

Cisplatin is an effective chemotherapeutic agent whose clinical use is limited by dose-dependent off-target toxicities, particularly hepatic and cardiac injury. The present study investigated the protective efficacy of rutin-mediated selenium nanoparticles (RUT-SeNPs) against cisplatin-induced hepato-cardiotoxicity in rats and compared their effects against those of free rutin and sodium selenite. RUT-SeNPs were characterized using dynamic light scattering, zeta potential analysis, transmission electron microscopy, X-ray diffraction, and UV–visible spectroscopy. Thirty-five male rats were randomly assigned to five groups: control (CON), cisplatin (CIS), CIS + Rutin, CIS + selenium, and CIS + RUT-SeNPs. Cisplatin administration caused marked hepatic and cardiac injury, as evidenced by altered liver-function indices, elevated cardiac injury biomarkers, oxidative stress, inflammatory activation, endoplasmic reticulum stress, and apoptosis. These effects were associated with increased lipid peroxidation, depletion of endogenous antioxidants, KEAP1 upregulation, Nrf2 suppression, activation of the TLR4/MAPK/NF-κB inflammatory axis, elevated TNF-α, IL-6, and COX-2 levels, and increased mRNA expression of the ER stress-related genes PERK, ATF4, ATF6, and CHOP. Cisplatin also promoted mitochondrial apoptosis, as indicated by increased Bax expression and cytochrome c release together with reduced Bcl-2 levels. Although rutin and sodium selenite partially mitigated these alterations, RUT-SeNPs produced the most pronounced protective effects by restoring redox homeostasis, suppressing inflammatory signaling, reducing the elevated expression of ER stress-related genes, and limiting mitochondrial apoptotic activation. These molecular improvements were accompanied by substantial preservation of the hepatic and cardiac histoarchitecture. Collectively, these results indicate that RUT-SeNPs may represent a promising nanoformulation for reducing cisplatin-induced hepato-cardiac toxicity through coordinated modulation of oxidative stress, inflammation, endoplasmic reticulum stress, and apoptosis.

## 1. Introduction

Cisplatin is still considered a cornerstone chemotherapeutic agent in the treatment of many solid tumors, but its clinical efficacy has been greatly impaired by significant dose limiting toxicities that are not restricted to the kidney but extend to include vital organs like the liver and heart [1]. Hepatotoxicity induced by cisplatin is characterized by hepatic cellular damage, disturbances in hepatic enzyme equilibrium, mitochondrial injury, as well as inflammatory and apoptotic cascades [2]. At the same time, cardiotoxicity induced by cisplatin is associated with oxidative injury to cardiac tissue, inflammatory tissue infiltration, dysfunction of cardiac contraction, and cardiomyocyte apoptosis. Overproduction of reactive oxygen species, impoverishment of endogenous antioxidant mechanisms, mitochondrial dysfunction, and continued expression of pro-inflammatory transcription factors are the major factors contributing to these adverse effects, and eventual structural and functional loss of hepatic and cardiac tissues [3].

In this regard, more focus has been paid to natural bioactive compounds as adjunctive approaches to prevent chemotherapy-induced organ toxicity. Rutin is a naturally occurring flavonoid glycoside with strong antioxidant, anti-inflammatory, and anti-apoptotic effects, although it does not have good bioavailability due to rapid metabolism [4]. The eco-synthesis of selenium nanoparticles with rutin as a bio-reducing and stabilizing agent is one of the most advanced nanotherapeutic approaches, where the necessary redox-modulating properties of selenium arecombined with the biological activity of rutin [5,6].

Although cisplatin-induced hepatic and cardiac toxicities have been documented, they have received considerably less mechanistic attention than cisplatin-associated nephrotoxicity, ototoxicity, and neurotoxicity [7]. Consequently, the molecular events responsible for the simultaneous development of hepatic and myocardial injury remain incompletely characterized [8]. Available evidence indicates that excessive reactive oxygen species generation, mitochondrial dysfunction, inflammatory signaling, endoplasmic reticulum stress, and apoptosis contribute to cisplatin-induced injury in both organs [9]. However, these processes are frequently examined individually or in a single tissue, leaving the coordinated molecular basis of cisplatin-induced cardiohepatic toxicity insufficiently defined.

This knowledge gap extends to the development of protective interventions. Rutin possesses antioxidant, anti-inflammatory, and anti-apoptotic activities [10], but its therapeutic application may be constrained by limited oral bioavailability and rapid metabolism. Selenium is an essential component of several redox-regulating selenoproteins; however, the biological activity and safety of selenium are strongly influenced by its chemical form and dose [11]. The synthesis of selenium nanoparticles using rutin as a reducing and stabilizing agent therefore provides a rationale for combining the complementary redox-modulating properties of selenium with the biological activity of rutin. Nevertheless, it remains unclear whether rutin-mediated selenium nanoparticles can provide coordinated protection against cisplatin-induced hepatic and cardiac injury or whether they offer greater efficacy than free rutin or inorganic sodium selenite.

To the best of our knowledge, the present study is the first in vivo investigation to evaluate RUT-SeNPs in a model of concurrent cisplatin-induced hepatic and cardiac injury while directly comparing their protective effects against those of free rutin and sodium selenite. The originality of this work lies in its integrated assessment of functional biomarkers, redox homeostasis, inflammatory signaling, ER-stress-associated transcripts, mitochondrial apoptotic mediators, histopathology, and NF-κB immunoreactivity in both organs. We hypothesized that RUT-SeNPs would provide broader cardiohepatic protection than their individual comparators through coordinated modulation of these interconnected molecular processes.

## 2. Results

### 2.1. Physicochemical Assessment of Selenium Nanoparticles Biosynthesized Using Rutin

#### 2.1.1. Dynamic Light Scattering and Zeta Potential Assessments of RUT-SeNPs

**Figure 1:**
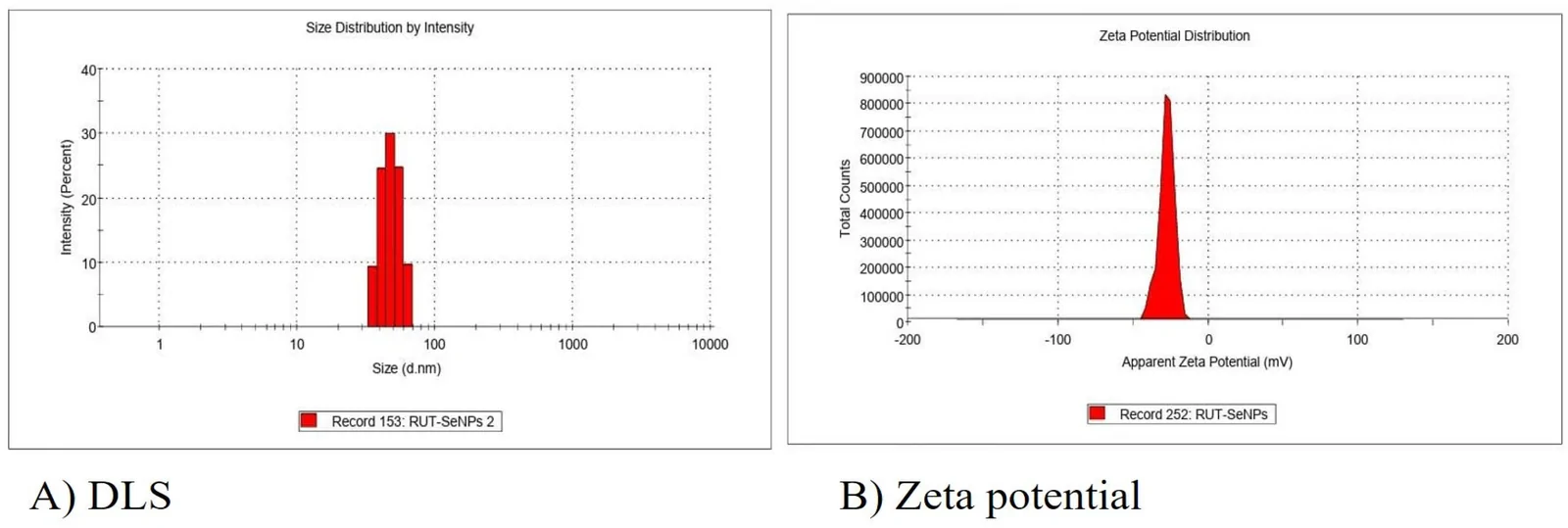
Physicochemical characterization of rutin-mediated selenium nanoparticles (RUT-SeNPs). (**A**) Dynamic light scattering (DLS); (**B**) Zeta potential distribution.

Dynamic light scattering (DLS) analysis demonstrated that the synthesized RUT-SeNPs exhibited a relatively narrow and unimodal size distribution, with an average hydrodynamic diameter in the nanoscale range of 80 nm, indicating homogeneous particle dispersion. The zeta potential measurement revealed a negative surface charge of approximately −25 mV, reflecting adequate electrostatic repulsion among particles and suggesting good colloidal stability of the nanosuspension (Figure 1).

#### 2.1.2. Transmission Electron Microscope Imaging of RUT-SeNPs

**Figure 2:**
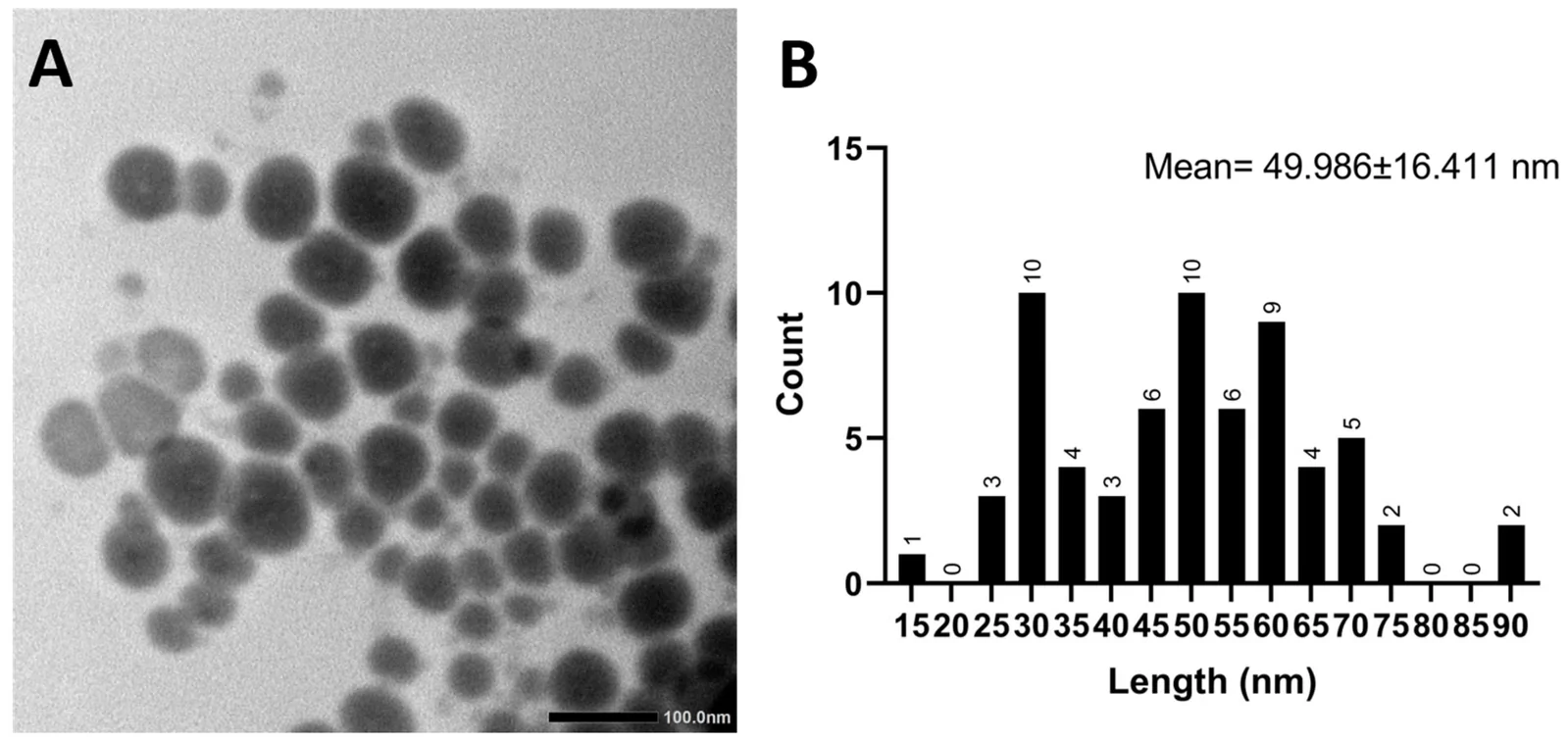
Transmission electron micrograph of rutin-mediated selenium nanoparticles (RUT-SeNPs) (**A**) Original image; (**B**) Quantitative analysis of 65 counted NPs. Scale bar = 100 nm.

Transmission electron microscopy (TEM) further confirmed the successful formation of selenium nanoparticles, revealing predominantly spherical particles with uniform morphology and minimal aggregation, with a mean particle size of 49.986 ± 16.411 nm. The observed particle size was consistent with the nanoscale range, although slightly smaller due to the absence of the hydration layer in the TEM measurements (Figure 2).

The smaller particle size observed via TEM (49.986 ± 16.411 nm) compared to the hydrodynamic diameter measured via DLS (~80 nm) may be attributed to differences between the two analytical techniques. TEM measures the dry particle core, whereas DLS reflects the hydrodynamic diameter of nanoparticles in suspension, including the surrounding hydration/capping layer. In addition, minor nanoparticle aggregation or agglomeration in the dispersed state may contribute to the larger apparent size detected by DLS.

#### 2.1.3. X-Ray Diffraction Analysis of RUT-SeNPs

**Figure 3:**
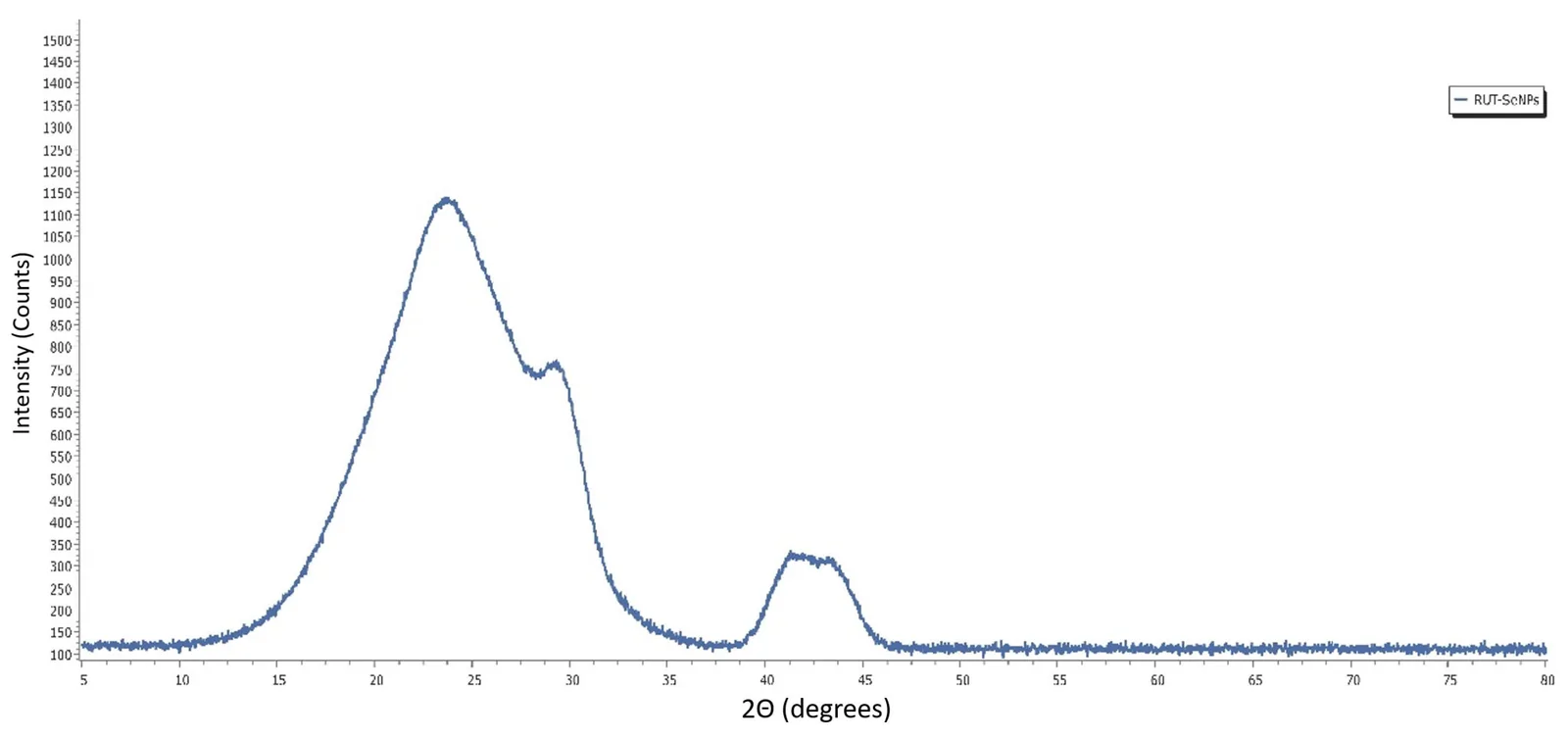
X-ray diffraction (XRD) pattern of rutin-mediated selenium nanoparticles (RUT-SeNPs).

The X-ray diffraction (XRD) pattern of rutin-mediated selenium nanoparticles (RUT-SeNPs) showed a broad, intense diffraction band centered at approximately $2\theta \;=\;24^{{}^{\circ}}$, together with less intense broad peaks around 29° and 41–43°. The broad nature of the recorded peaks indicates that the synthesized nanoparticles possess a predominantly amorphous or poorly crystalline structure. This pattern may be attributed to the nanoscale form of selenium and the presence of rutin-derived phytochemical capping molecules on the nanoparticle surface (Figure 3).

#### 2.1.4. UV–Visible Spectroscopy

**Figure 4:**
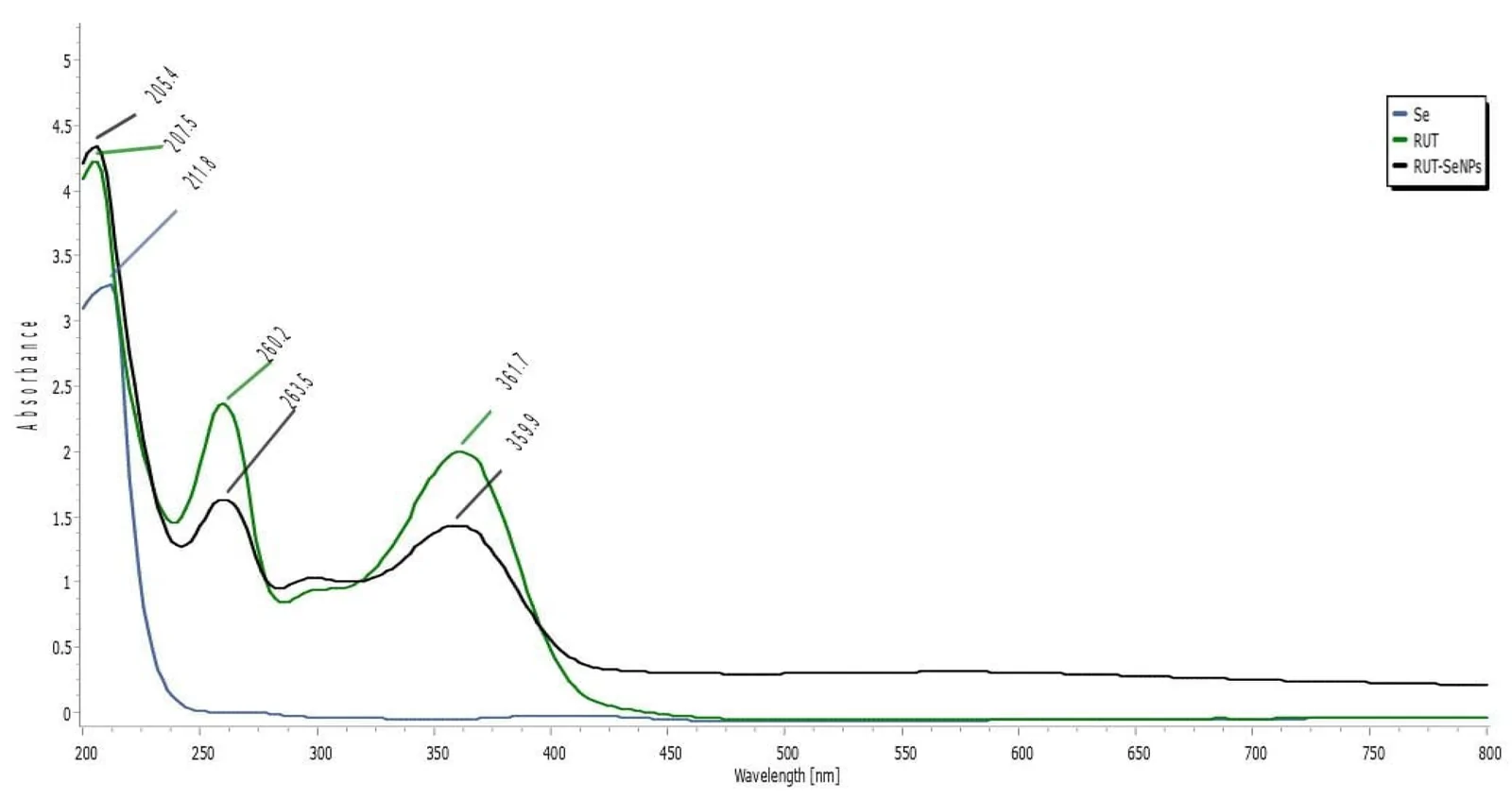
UV–visible spectra of rutin (RUT) and rutin-mediated selenium nanoparticles (RUT-SeNPs).

UV–visible spectroscopy was used to track the optical behaviors of selenium and rutin before and after nanoparticle formation. Pure rutin displayed its typical absorption bands in the UV region, reflecting its intrinsic flavonoid chromophore system. After the synthesis of RUT-SeNPs, clear alterations in absorbance intensity along with slight shifts in the characteristic peaks were observed. In addition, a broader absorption feature appeared, indicating changes in the electronic environment due to nanoparticle formation. These spectral variations confirm the successful conversion of selenium ions into nanosized particles and highlight the interaction between selenium and the functional groups of rutin (Figure 4).

### 2.2. Evaluation of Liver Function Biomarkers

**Figure 5:**
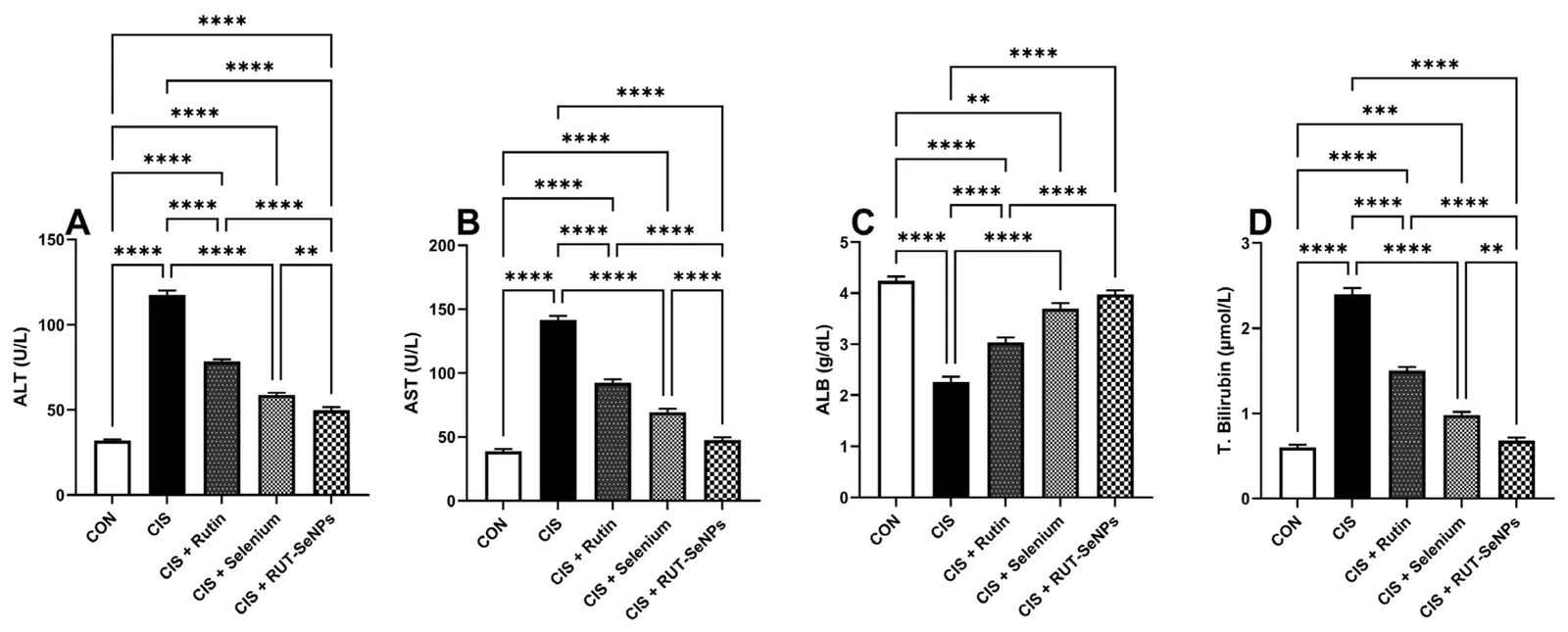
Effects of rutin-mediated selenium nanoparticles (RUT-SeNPs), rutin, and sodium selenite on cisplatin-induced alterations in hepatic function biomarkers. (**A**) Serum alanine aminotransferase (ALT) (**B**) aspartate aminotransferase (AST), (**C**) albumin (ALB), and (**D**) total bilirubin levels were assessed in control (CON), cisplatin-treated (CIS), and cisplatin-treated rats receiving rutin, selenium, or RUT-SeNPs. Data are expressed as mean ± standard error (*n* = 7). Statistical analysis was performed using one-way ANOVA followed by Tukey’s post hoc test. ** *p* < 0.01, *** *p* < 0.001, and **** *p* < 0.0001.

As shown in Figure 5, hepatic function underwent remarkable degradation when cisplatin was used, which was evidenced by the significant increases in serum ALT, AST, and total bilirubin levels, alongside a significant decrease in albumin concentration, compared to the control group (*p* < 0.05). The presence of these biochemical changes indicates the breakage of hepatocellular membranes and the inability to synthesize, which proves the hepatotoxicity of cisplatin exposure.

These disturbances in hepatic function were greatly alleviated by therapeutic treatment with sodium selenite or rutin, which also partly returned liver enzyme activities and albumin levels to normal levels. Notably, molecules that had the most significant protective effect were the treatment with rutin-mediated selenium nanoparticles (RUT-SeNPs) had the most significant protective effect.

### 2.3. Serum Cardiac Injury Biomarkers (CK-MB, cTnT, FABP3, MYL3, and ANP)

**Figure 6:**
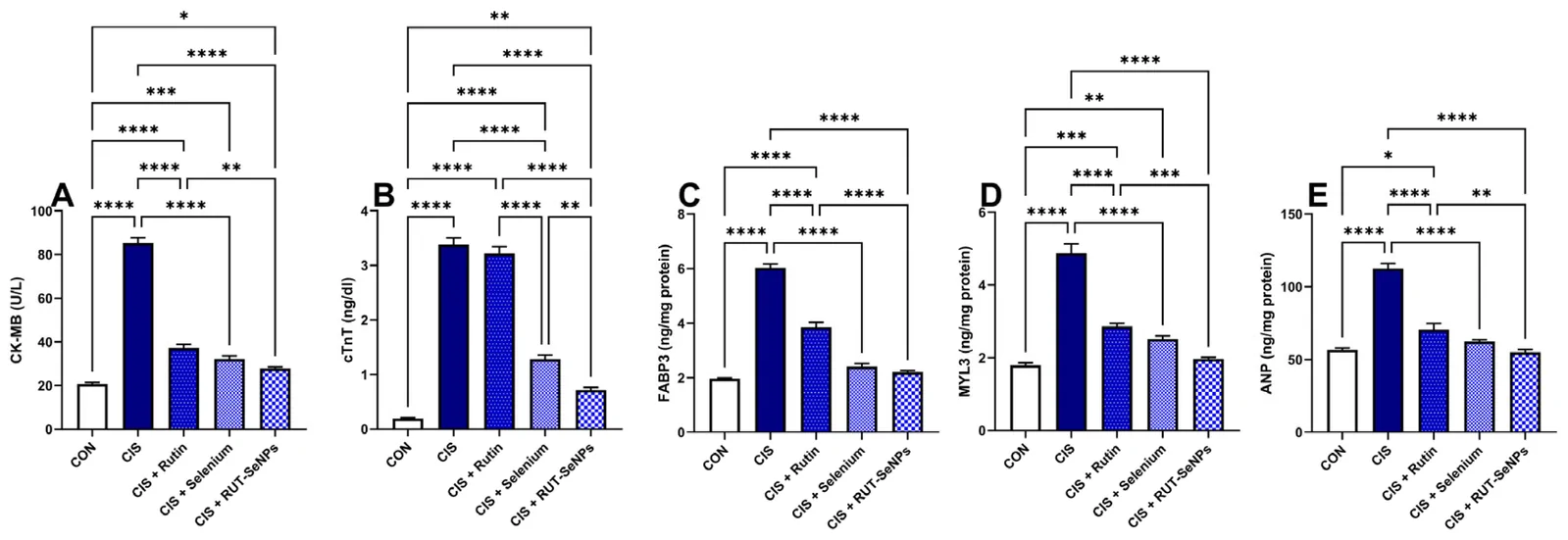
Effects of rutin-mediated selenium nanoparticles (RUT-SeNPs), rutin, and sodium selenite on cisplatin-induced alterations in serum cardiac injury biomarkers. (**A**) Serum creatine kinase-MB (CK-MB), (**B**) cardiac troponin T (cTnT), (**C**) heart-type fatty acid binding protein (FABP3), (**D**) myosin light chain-3 (MYL3), and (**E**) atrial natriuretic peptide (ANP) levels were evaluated in control (CON), cisplatin-treated (CIS), and cisplatin-treated rats receiving rutin, selenium, or RUT-SeNPs. Data are represented as mean ± standard error (SEM) (*n* = 7). Statistical significance was assessed using one-way ANOVA followed by Tukey’s post hoc test. * *p* < 0.05, ** *p* < 0.01, *** *p* < 0.001, and **** *p* < 0.0001.

As demonstrated in Figure 6, exposure to cisplatin induced a significant rise in the level of circulating cardiac injury markers, including CK-MB, cTnT, FABP3, MYL3, and ANP, compared to the control group (*p* < 0.05). The increased levels of these biomarkers means that there is a large amount of myocardial damage, loss of membrane integrity, and functional cardiac stress due to cardiotoxicity caused by cisplatin.

Sodium selenite or rutin administration had a significant effect on cisplatin-induced upregulation of these cardiac markers, indicating partial recovery of myocardial integrity. Notably, the strongest cardioprotective effect was observed in the case of treatment with rutin-mediated selenium nanoparticles (RUT-SeNPs), which could effectively decrease serum CK-MB, cTnT, FABP3, MYL3, and ANP levels to physiological levels.

### 2.4. Evaluation of Hepatic and Cardiac MDA Marker, and Keap-1/Nrf2 Pathway

**Figure 7:**
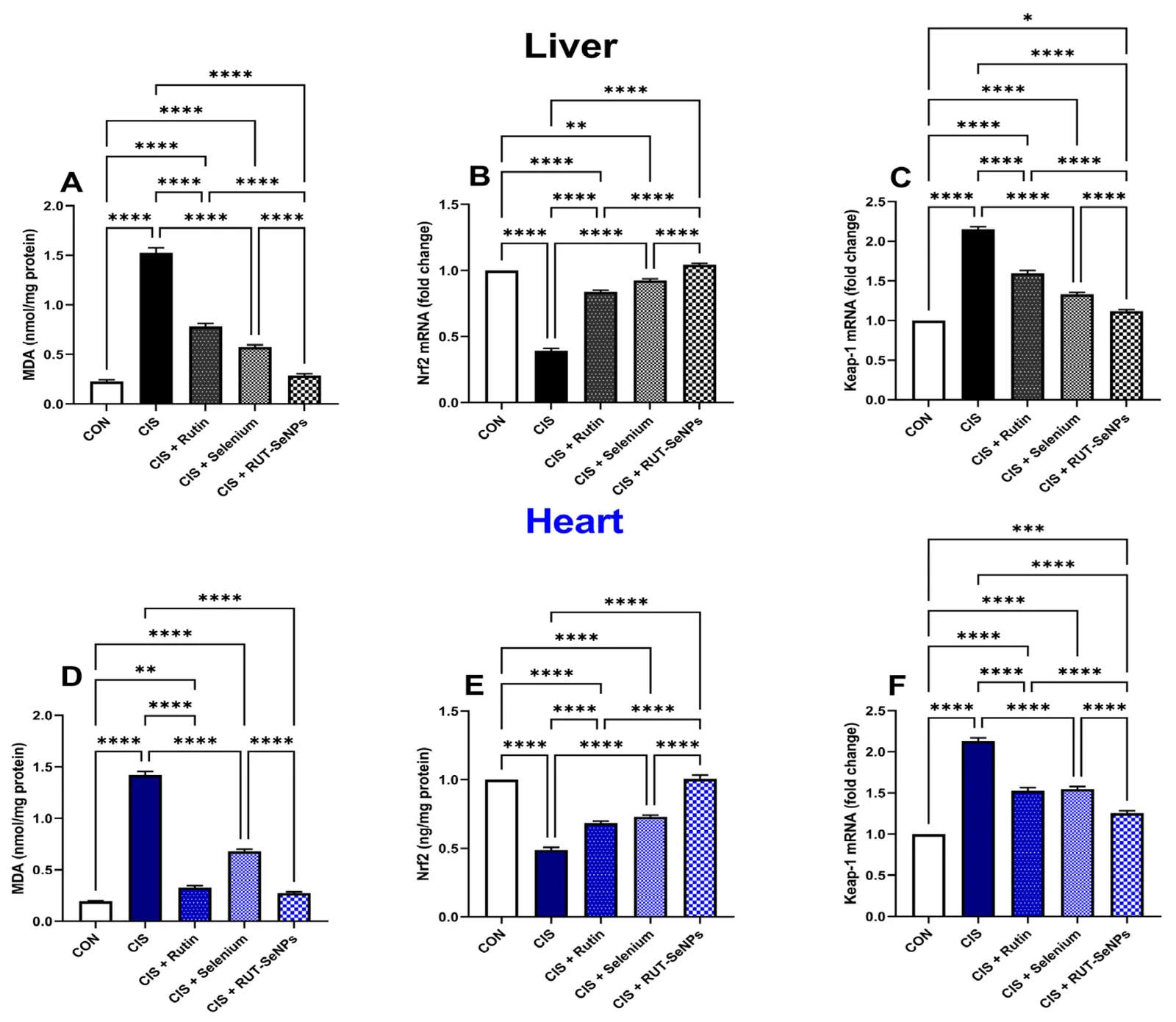
Effects of rutin-mediated selenium nanoparticles (RUT-SeNPs), rutin, and sodium selenite on cisplatin-induced alterations in oxidative stress and Keap-1/Nrf2 antioxidant signaling in hepatic and cardiac tissues. (**A**) Hepatic malondialdehyde (MDA) levels, (**B**) hepatic Nrf2 mRNA expression, and (**C**) hepatic Keap-1 mRNA expression, as well as (**D**) cardiac MDA levels, (**E**) cardiac Nrf2 mRNA expression, and (**F**) cardiac Keap-1 mRNA expression, were evaluated in control rats (CON), cisplatin-treated rats (CIS), and cisplatin-treated rats receiving rutin, sodium selenite, or RUT-SeNPs. Data were presented as mean ± standard error (*n* = 7). Statistical significance was determined using one-way ANOVA followed by Tukey’s post hoc test. * *p* < 0.05, ** *p* < 0.01, *** *p* < 0.001, and **** *p* < 0.0001.

As shown in Figure 7, cisplatin administration induced marked oxidative stress in both hepatic and cardiac tissues. In the liver, cisplatin significantly elevated MDA levels, suppressed Nrf2 mRNA expression, and increased Keap-1 mRNA expression compared to the control group. Similarly, cardiac tissues of cisplatin-treated rats exhibited significantly increased MDA levels and Keap-1 mRNA expression, accompanied by a significant reduction in Nrf2 levels. These findings indicate enhanced lipid peroxidation and impairment of the Keap-1/Nrf2-regulated antioxidant defense system following cisplatin exposure.

Treatment with rutin or sodium selenite significantly ameliorated the cisplatin-induced oxidative alterations in both tissues, as demonstrated by reduced MDA and Keap-1 levels together with partial recovery of Nrf2. Notably, treatment with RUT-SeNPs exerted the most prominent protective effect, markedly reducing hepatic and cardiac MDA accumulation, suppressing Keap-1 upregulation, and restoring Nrf2 levels to control values.

### 2.5. Antioxidant Defense Markers (GSH, GR, GPx, SOD, and CAT)

**Figure 8:**
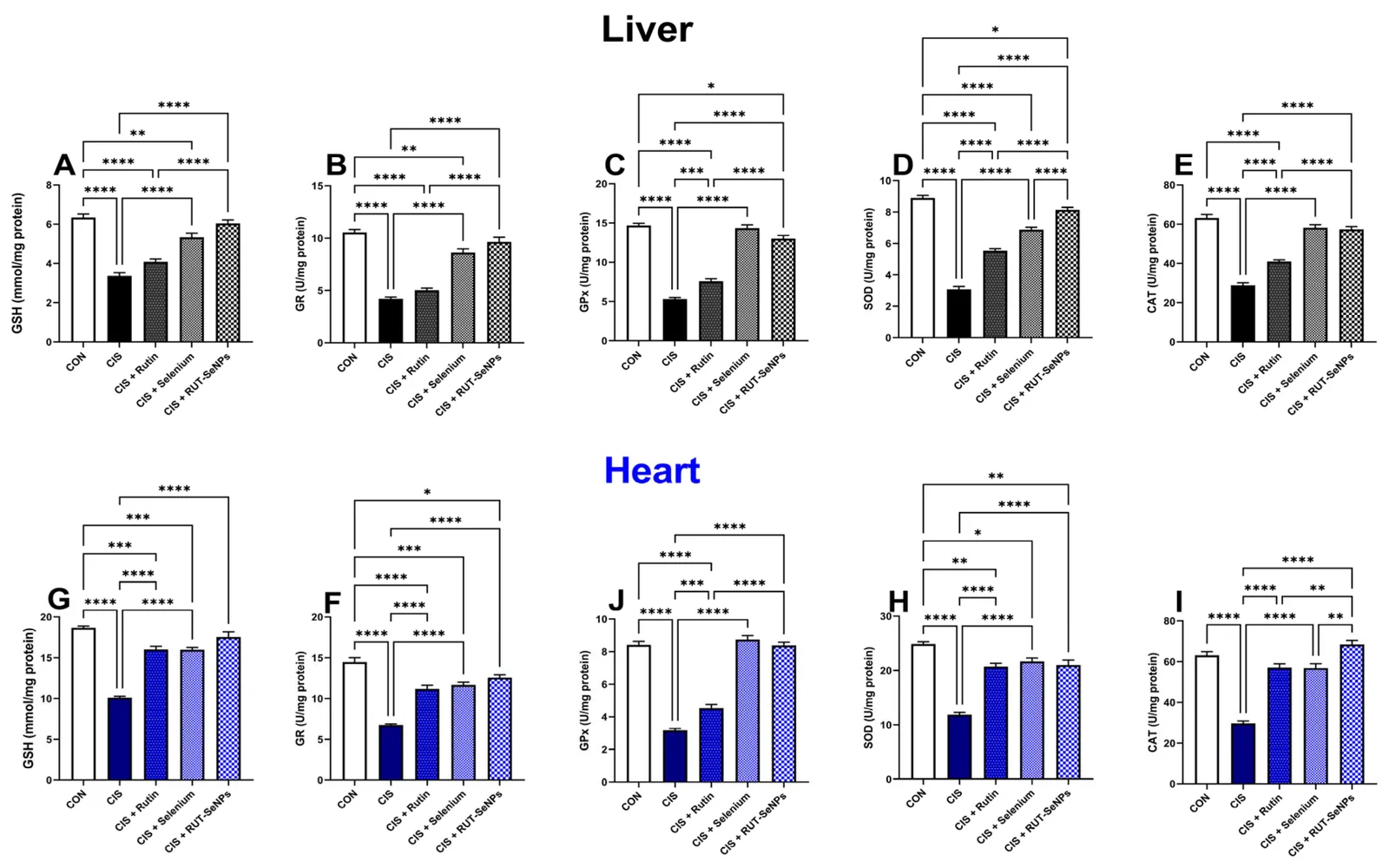
Effects of rutin-mediated selenium nanoparticles (RUT-SeNPs), rutin, and sodium selenite on cisplatin-induced alterations in antioxidant defense markers in hepatic and cardiac tissues. Hepatic (**A**) reduced glutathione (GSH), (**B**) glutathione reductase (GR), (**C**) glutathione peroxidase (GPx), (**D**) superoxide dismutase (SOD), and (**E**) catalase (CAT) levels were assessed, along with corresponding (**G**) cardiac GSH, (**F**) GR, (**J**) GPx, (**H**) SOD, and (**I**) CAT activities in control (CON), cisplatin-treated (CIS), and cisplatin-treated rats receiving rutin, selenium, or RUT-SeNPs. Data are presented as mean ± standard error (*n* = 7). Statistical analysis was performed using one-way ANOVA followed by Tukey’s post hoc test. * *p* < 0.05, ** *p* < 0.01, *** *p* < 0.001, and **** *p* < 0.0001.

The antioxidant defense system of hepatic and cardiac tissues was also seriously impaired by cisplatin administration, as the reduced glutathione (GSH) content and the activities of glutathione reductase (GR), glutathione peroxidase (GPx), superoxide dismutase (SOD), and catalase (CAT) were significantly reduced in cisplatin-treated tissues compared to the control group (Figure 8, *p* < 0.05). These changes represent the deep dysfunction of enzymatic and non-enzymatic antioxidant systems, which are signs of the severe oxidative load caused by exposure to cisplatin.

Sodium selenite or rutin co-treatment had a significant effect in alleviating these disturbances (*p* < 0.05) compared to the CIS group, partly recovering the antioxidant enzyme activities and GSH levels of liver and heart tissues. It is worth noting that rutin-mediated selenium nanoparticle (RUT-SeNPs) administration had the strongest protective effect, and antioxidant parameters were almost close to normal levels.

### 2.6. Inflammatory Markers (TNF-α, NF-κB, IL-6, and COX-2)

**Figure 9:**
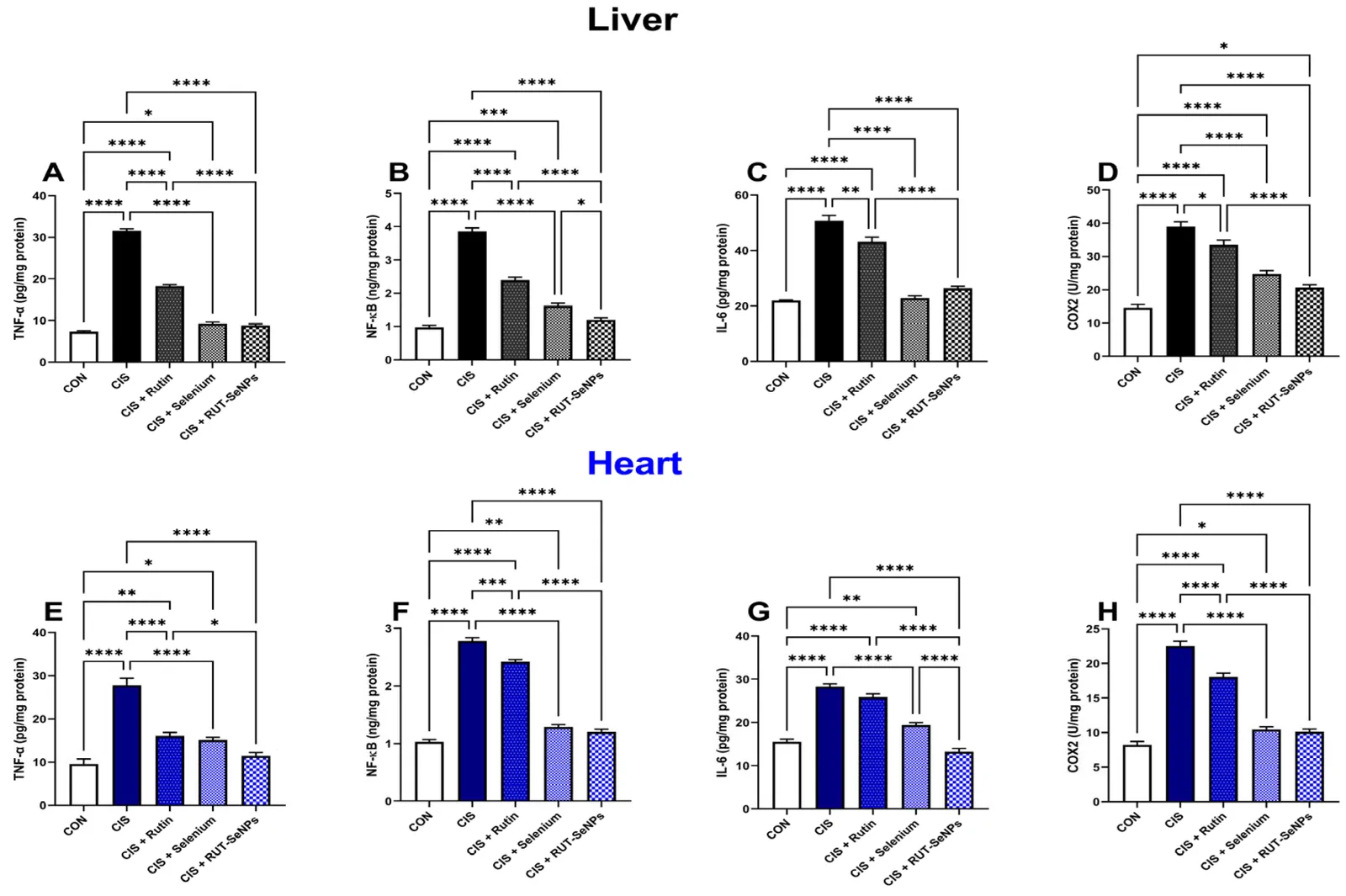
Effects of rutin-mediated selenium nanoparticles (RUT-SeNPs), rutin, and sodium selenite on cisplatin-induced inflammatory markers in hepatic and cardiac tissues. Hepatic (**A**) tumor necrosis factor-α (TNF-α), (**B**) nuclear factor kappa B (NF-κB), (**C**) interleukin-6 (IL-6), and (**D**) cyclooxygenase-2 (COX-2) levels were evaluated, along with corresponding cardiac (**E**) TNF-α, (**F**) NF-κB, (**G**) IL-6, and (**H**) COX-2 expression in control (CON), cisplatin-treated (CIS), and cisplatin-treated rats receiving rutin, selenium, or RUT-SeNPs. Data were expressed as mean ± standard error (SEM) (*n* = 7). Statistical analysis was performed using one-way ANOVA followed by Tukey’s post hoc test. * *p* < 0.05, ** *p* < 0.01, *** *p* < 0.001, and **** *p* < 0.0001.

As illustrated in Figure 9, both hepatic and cardiac tissue responses to cisplatin exposure varied strongly in the form of high TNF-α, NF-kB, IL-6 and COX-2 levels compared to the control group (*p* < 0.05). These changes indicate the stimulation of canonical pro-inflammatory signaling and an increase in the transcription of downstream inflammatory mediators, which validates the significant inflammatory injury caused by cisplatin treatment.

Treatment with sodium selenite or rutin greatly affected the suppression of this inflammatory activation through the expression of TNF-α, NF-kB, IL-6, and COX-2 in liver and cardiac tissues. Notably, the best protective effect against inflammation was provided by treatment with rutin-mediated selenium nanoparticles (RUT-SeNPs), which brought the levels of inflammatory mediators close to baseline.

### 2.7. MAPK, TLR4, and Cytochrome C Signaling in Hepatic and Cardiac Tissues

**Figure 10:**
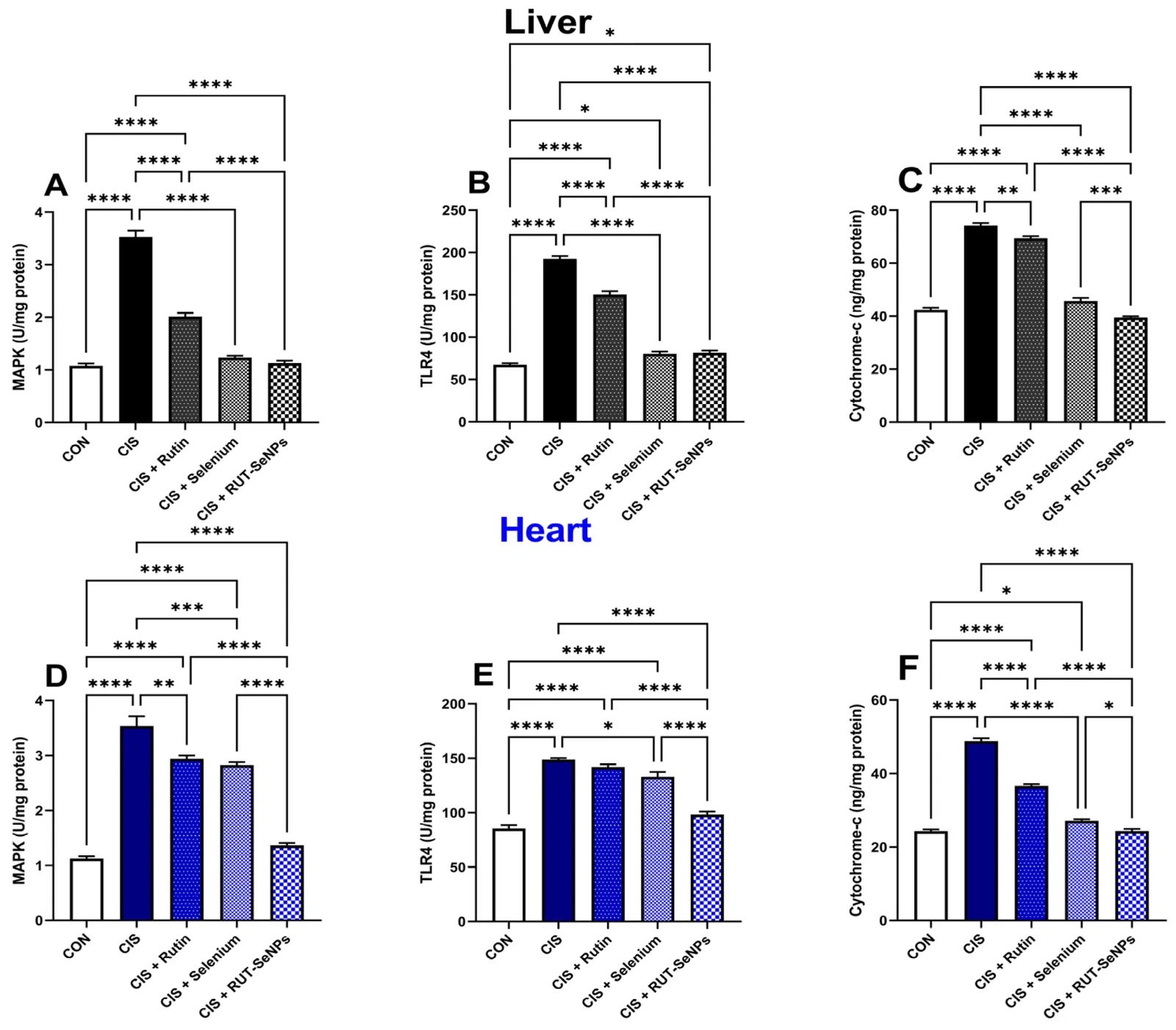
Effects of rutin, sodium selenite, and rutin-mediated selenium nanoparticles (RUT-SeNPs) on cisplatin-induced alterations in MAPK, TLR4, and cytochrome c levels in hepatic and cardiac tissues. Hepatic levels of (**A**) mitogen-activated protein kinase (MAPK), (**B**) Toll-like receptor 4 (TLR4), and (**C**) cytochrome c, as well as cardiac levels of (**D**) MAPK, (**E**) TLR4, and (**F**) cytochrome c, were assessed in control rats (CON), cisplatin-treated rats (CIS), and cisplatin-treated rats receiving rutin, sodium selenite, or RUT-SeNPs. Data are presented as mean ± standard error (SEM) (*n* = 7). Statistical analysis was performed using one-way ANOVA followed by Tukey’s post hoc test. * *p* < 0.05, ** *p* < 0.01, *** *p* < 0.001, and **** *p* < 0.0001.

As illustrated in Figure 10, cisplatin administration markedly activated stress-, inflammation-, and apoptosis-associated signaling in both hepatic and cardiac tissues. In the liver, cisplatin significantly increased MAPK and TLR4 levels compared to the control group, indicating activation of cellular stress and innate inflammatory signaling. Moreover, hepatic cytochrome c levels were markedly elevated, suggesting mitochondrial membrane disruption and initiation of mitochondria-dependent apoptotic events. Treatment with rutin reduced these alterations to varying degrees; however, hepatic MAPK, TLR4, and cytochrome c levels remained higher than those observed in animals receiving selenium or RUT-SeNPs. Sodium selenite substantially attenuated cisplatin-induced hepatic signaling disturbances, while RUT-SeNPs exerted the most pronounced protective effect, reducing MAPK, TLR4, and cytochrome c levels close to control values.

A similar pattern of injury was observed in cardiac tissues, where cisplatin significantly increased MAPK, TLR4, and cytochrome c levels relative to the control group. These findings reflect the induction of myocardial cellular stress, inflammatory pathway activation, and mitochondrial apoptotic signaling following cisplatin exposure. Although rutin and sodium selenite significantly ameliorated several of these changes, their modulatory effects on cardiac MAPK and TLR4 were less prominent than those produced by RUT-SeNPs. Notably, RUT-SeNPs markedly suppressed the cisplatin-induced increases in cardiac MAPK, TLR4, and cytochrome c levels, with values approaching those of the control group. These results indicate that RUT-SeNPs provide hepato-cardioprotective activity through coordinated inhibition of MAPK/TLR4-mediated stress and inflammatory responses, together with suppression of mitochondrial cytochrome c release.

### 2.8. Apoptotic Gene Expression (BAX and BCL-2)

**Figure 11:**
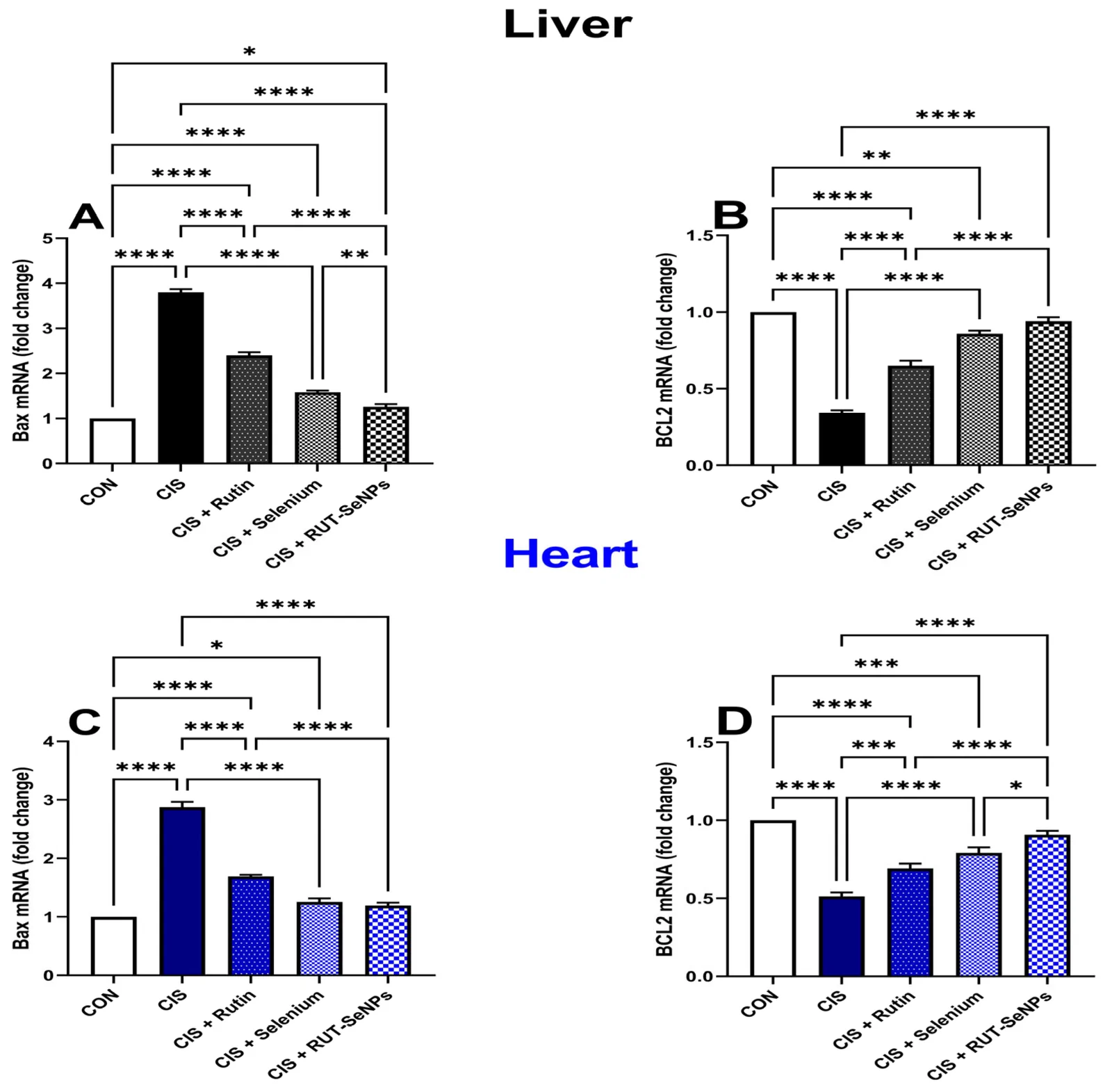
Effects of rutin-mediated selenium nanoparticles (RUT-SeNPs), rutin, and sodium selenite on cisplatin-induced modulation of apoptotic gene expression in hepatic and cardiac tissues. Hepatic (**A**) *Bax* mRNA expression and (**B**) *BCL-2* mRNA expression, as well as cardiac (**C**) *Bax* and (**D**) *BCL-2* transcript levels, were evaluated in control (CON), cisplatin-treated (CIS), and cisplatin-treated rats receiving rutin, selenium, or RUT-SeNPs. Gene expression levels were normalized to β-actin and expressed as fold change relative to the control group. Data are presented as mean ± standard error (SEM) (*n* = 7). Statistical significance was determined using one-way ANOVA followed by Tukey’s post hoc test. * *p* < 0.05, ** *p* < 0.01, *** *p* < 0.001, and **** *p* < 0.0001.

Exposure to cisplatin caused a great disturbance in hepatic and cardiac tissue apoptotic homeostasis, as shown by the significant upregulation of the pro-apoptotic gene BAX and a significant decrease in the expression of the anti-apoptotic gene BCL-2 relative to the control group, as shown in Figure 11 (*p* < 0.05). This BAX/BCL-2 ratio imbalance indicates the activation of the intrinsic mitochondrial apoptotic pathway and highlights the role played by programmed cell death in tissue damage caused by cisplatin.

The addition of sodium selenite or rutin improved these molecular perturbations (*p* < 0.05 vs. CIS group) by suppressing BAX expression and restoring the transcriptions of BCL-2. Notably, the treatment with rutin-mediated selenium nanoparticles (RUT-SeNPs), exhibited the strongest regulating effect, restoring the apoptotic balance to almost physiological levels.

### 2.9. Expression of ER Stress-Related Genes in Hepatic and Cardiac Tissues

**Figure 12:**
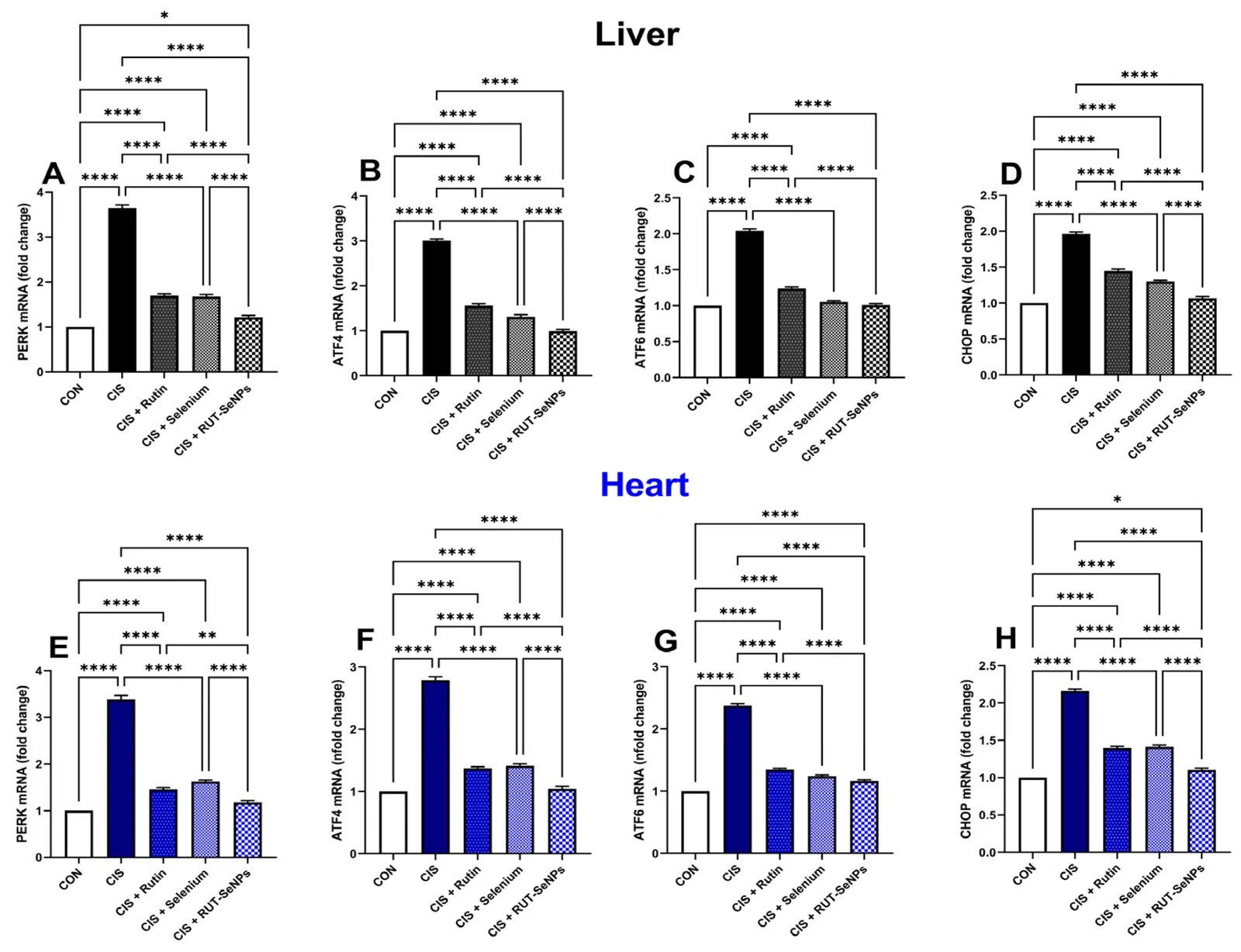
Effects of rutin, sodium selenite, and rutin-mediated selenium nanoparticles on cisplatin-induced changes in ER stress-related gene expression in hepatic and cardiac tissues. Hepatic mRNA expression levels of (**A**) protein kinase RNA-like endoplasmic reticulum kinase (PERK), (**B**) activating transcription factor 4 (ATF4), (**C**) activating transcription factor 6 (ATF6), and (**D**) C/EBP homologous protein (CHOP), as well as cardiac mRNA expression levels of (**E**) PERK, (**F**) ATF4, (**G**) ATF6, and (**H**) CHOP, were evaluated in control rats (CON), cisplatin-treated rats (CIS), and cisplatin-treated rats receiving rutin, sodium selenite, or RUT-SeNPs. Data presented as mean ± standard error (SEM) (*n* = 7). Statistical significance was determined using one-way ANOVA followed by Tukey’s post hoc test. * *p* < 0.05, ** *p* < 0.01, and **** *p* < 0.0001.

As demonstrated in Figure 12, cisplatin administration significantly altered the transcriptional expression of ER stress-related genes in hepatic and cardiac tissues. In the liver, cisplatin significantly increased the mRNA expression levels of PERK, ATF4, ATF6, and CHOP compared to the control group. A comparable transcriptional response was observed in cardiac tissues, where cisplatin significantly upregulated all investigated ER stress-related genes. These findings demonstrate the increased expression of genes associated with the unfolded protein response; however, they do not directly establish activation of ER stress at the protein or functional level.

Treatment with rutin or sodium selenite significantly reduced the cisplatin-induced increases in PERK, ATF4, ATF6, and CHOP mRNA expression in both hepatic and cardiac tissues. RUT-SeNPs produced the greatest reductions in the expression of these genes, with values approaching those of the control group. Thus, RUT-SeNP administration was associated with marked modulation of the ER stress-related transcriptional response induced by cisplatin.

### 2.10. Histopathological and Immunohistochemical Analysis

**Figure 13:**
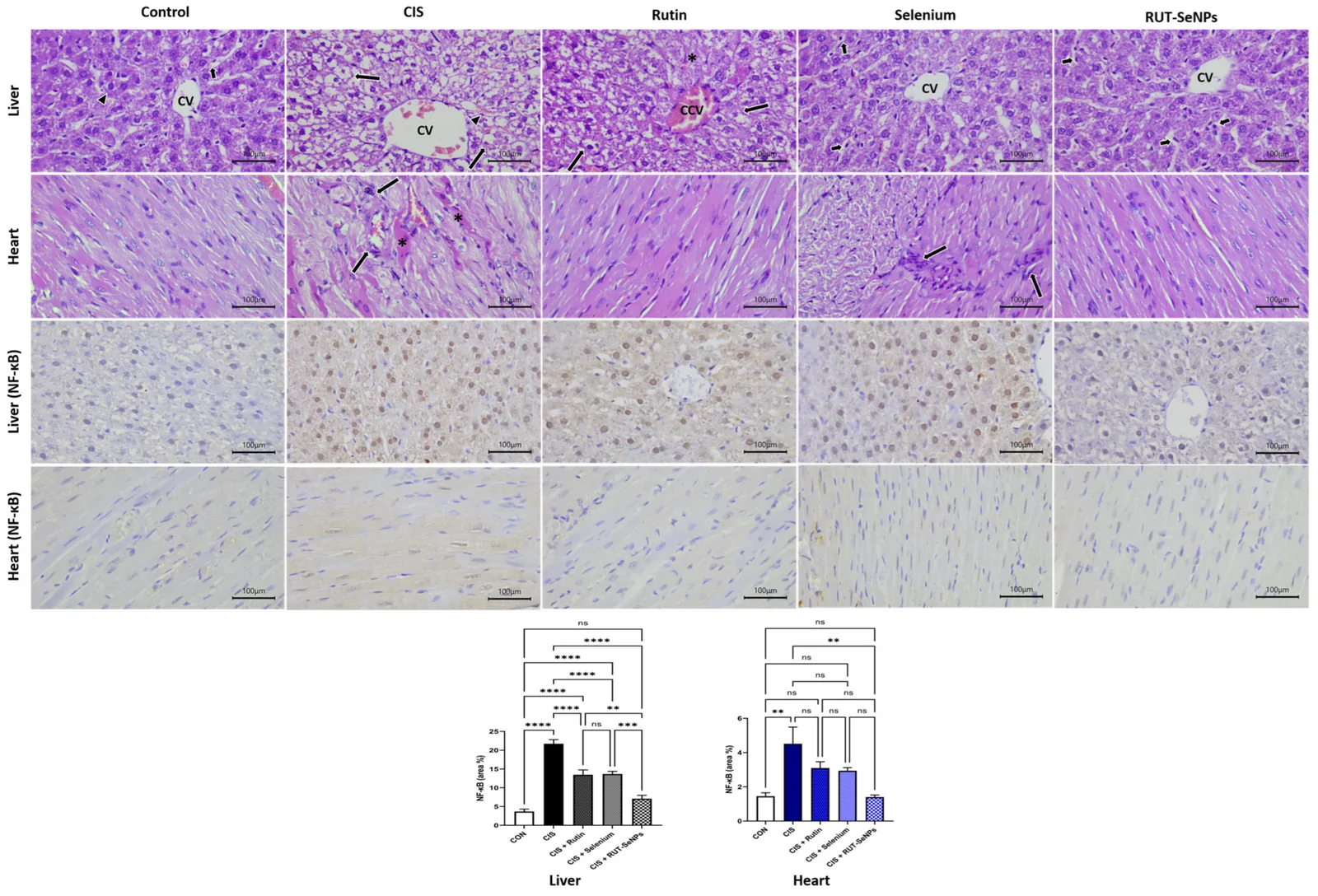
Photomicrographs of hepatic and cardiac tissues of all groups. Regarding hepatic tissues, the control group showed a normal hepatic lobule structure, with a central vein (CV), hepatocytes (short arrow), and Kupffer cells (arrow head) in sinusoids. The CIS group showed severe degenerated hepatocytes (long arrow) and interstitial hemorrhage (arrow head).The CIS + Rutin group showed a congested central vein (CCV), degenerated hepatocytes (long arrow), and focal necrosis (asterisk). Both the CIS + Selenium and CIS + RUT-SeNPs groups revealed an approximately normal central vein (CV) and hepatocytes with activated Kupffer cells (short arrow). Concerning cardiac tissues, the control group showed normally arranged cardiomyocytes. The CIS group showed highly acidophilic hyaline necrotic myocytes (asterisk) and inflammatory cellular infiltration (long arrow). Both the CIS + Rutin and CIS + RUT-SeNPs groups showed marked improved cardiomyocytes. Meanwhile, the CIS + Selenium group showed inflammatory cells infiltration (long arrow) (H&E, ×400). Regarding NF-κB immunostaining in hepatic tissues, both the control and CIS + RUT-SeNPs groups showed weak expression, whilethe CIS + Selenium group showed moderate expression, and both the CIS and CIS + Rutin groups showed marked expression. Concerning NF-κB immunostaining in cardiac tissues, all groups except the CIS group showed weak expression, while the CIS group showed mild expression. (DAB, ×400). Quantitative analysis of IHC area % for NF-κB is expressed as mean ± S.E.M (*n* = 7). Statistical significance was determined using one-way ANOVA followed by Tukey’s post hoc test. ns: non-significant, * *p* < 0.05, ** *p* < 0.01, *** *p* < 0.001, and **** *p* < 0.0001.

The hepatic tissues of the control group showed a hepatic lobule histological pattern with a central vein, radiating liver cords of hepatocytes with rounded open face nuclei and sinusoids between cords with guarding Kupffer cells. The CIS group revealed degeneration features with large swollen hepatocytes and interstitial hemorrhage. The CIS + Rutin group showed a congested central vein, focal necrotic area, and degenerated hepatocytes. Both the CIS + Selenium and CIS + RUT-SeNPs groups revealed nearly normal hepatic lobules except for increased numbers of activated Kupffer cells. The cardiac tissues of the control group showed normally arranged cardiomyocytes with central oval nuclei and acidophilic cytoplasm. The CIS group showed toxicity pattern with inflammatory cellular infiltration in the interstitial area and; increased acidophilia of the necrotic cardiomyocytes. Selenium group showed infiltration of inflammatory cells without a degeneration pattern. Both the CIS + Rutin and CIS + RUT-SeNPs groups showed marked improvement with approximately normally arranged cardiomyocytes. The hepatic tissues of both the control and CIS + RUT-SeNPs groups showed weak NF-κB expression, while the CIS and CIS + Rutin groups revealed marked expression, and the Selenium group exhibited moderate expression. The cardiac tissues of the CIS group revealed mild NF-κB expression; however the other groups showed weak staining (Figure 13). The quantitative analysis of NF-κB expression showed a remarkable increase in the CIS group compared to the Control and CIS + RUT-SeNPs groups (*p* < 0.001).

## 3. Discussion

Cisplatin remains one of the most efficient chemotherapeutic agents in solid tumors [12], but its therapeutic efficacy is often compromised by systemic toxicities that affect other non-target organs [1]. Although nephrotoxicity is the best-researched adverse effect, it is becoming clear through experimental evidence that other tissues can also fall victim to cisplatin toxicity, including hepatic and cardiac tissues [13]. This paper shows detailed biochemical, molecular, and histological evidence supporting the fact that cisplatin induces orchestrated oxidative, inflammatory, and apoptotic disruptions in liver and heart tissues [14]. Notably, selenium nanoparticles prepared usingrutin as a bio-reducing and stabilizing agent had better protective ability compared to sodium selenite or rutin used in isolation. These results highlight the focal importance of redox imbalance in cisplatin toxicity as well as the biological significance of selenium formulation and delivery in the regulation of the trace element dependent cytoprotective response [15].

Free rutin has previously been shown to reduce cisplatin-induced myocardial oxidative stress, inflammatory cytokine production, cardiac biomarker release, and histopathological injury [16]. Selenium and bioactive-functionalized SeNPs have also demonstrated protective effects in several cisplatin toxicity models, while rutin-modified SeNPs have been reported to attenuate oxidative neuronal injury through Nrf2/HO-1-associated responses. Furthermore, RUT-SeNPs have shown antioxidant, anti-inflammatory, and anti-apoptotic effects in neurological models unrelated to cisplatin [17,18]. The present study extends these observations by demonstrating the protective effects of RUT-SeNPs in the simultaneous hepatic and cardiac context of cisplatin toxicity. Importantly, the study integrates comparative evaluation against free rutin and sodium selenite with parallel assessment of KEAP-1/Nrf2-associated antioxidant defense, TLR4/MAPK/NF-κB-related inflammation, PERK/ATF4/ATF6/CHOP-associated ER stress, and Bax/Bcl-2/cytochrome c-related mitochondrial apoptosis in both organs. Thus, the novelty of the study lies in its dual-organ, comparative, and multi-pathway design rather than in the general antioxidant activity of rutin or selenium nanoparticles.

Increased levels of hepatic transaminases and bilirubin, accompanied by low albumin volumes, are clear indicator of disturbance in the integrity of hepatocellular membranes and synthetic dysfunction after exposure to cisplatin [19]. At the cellular level cisplatin can be hydrolyzed to form a reactive platinum complex that can react with nucleic acids, proteins and mitochondrial structures [20]. Mitochondria are especially important intracellular targets because when platinum is localized to mitochondria, it enhances the generation of excessive reactive oxygen species [21]. This ROS burst triggers cascades of lipid peroxidation, as the level of malondialdehyde in the tissue of the liver is elevated [22]. The resulting peroxidative damage of membrane phospholipids leads to the destruction of cell architecture and enhances the release of enzymes into the circulation proving the observed biochemical defects [23].

There is similar susceptibility in cardiac tissue. Raised serum CK-MB, cardiac troponin T, FABP3, MYL3, and ANP levels are signs of structural myocardial damage and contractile strain [24]. The high oxidative metabolism and network of mitochondria are particularly important in the myocardium, which is especially vulnerable to redox imbalances [25]. Oxidative overload caused by cisplatin interferes with the functioning of the mitochondrial electron transport chain, resulting in the inefficient production of ATP, and an increase in the production of superoxides [26]. This type of mitochondrial dysfunction is a probable cause of the observed release of myocardial biomarkers and functional impairment [27].

**Figure 14:**
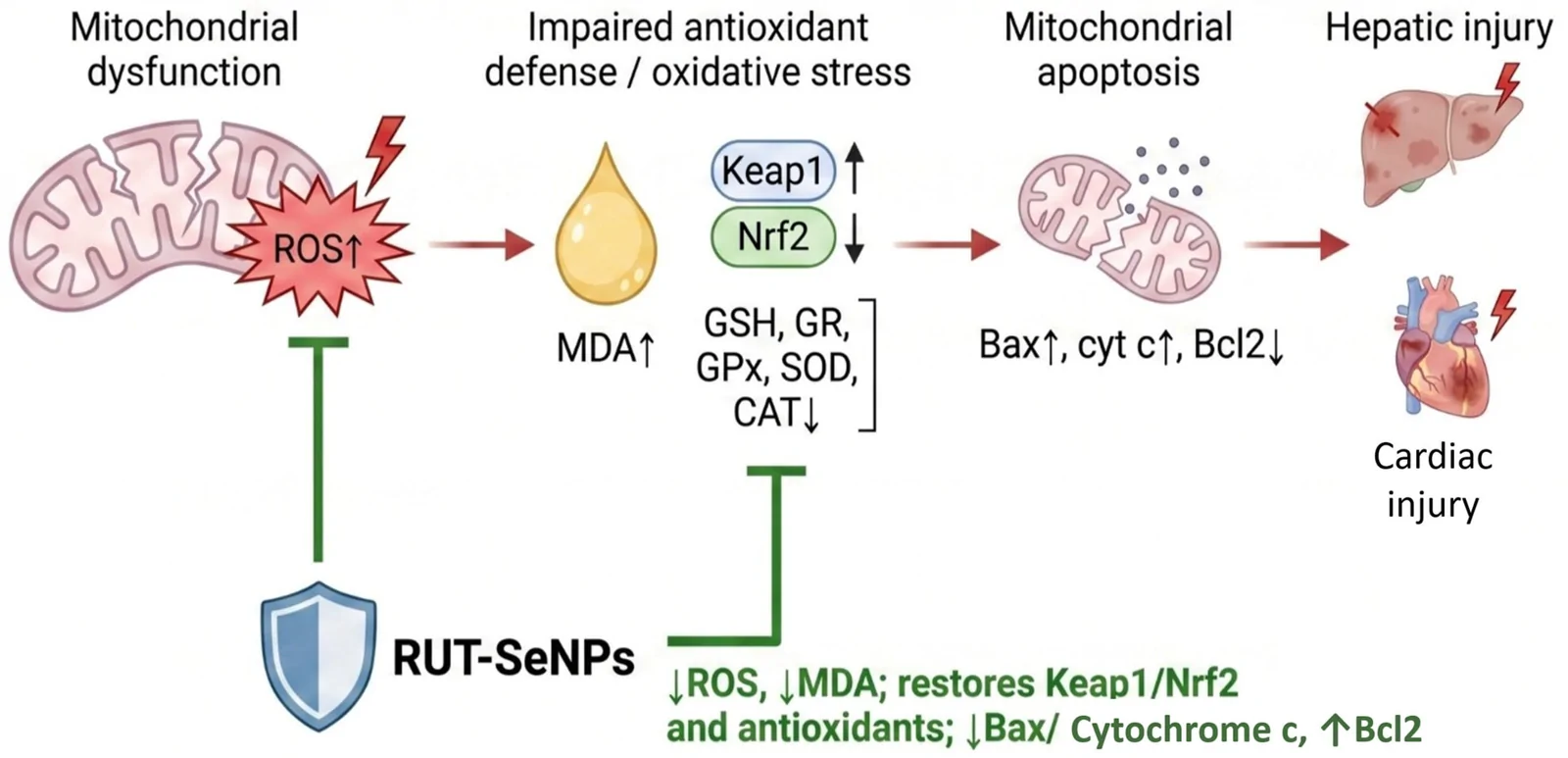
Proposed mechanistic model illustrating the protective effects of RUT-SeNPs against cisplatin-induced oxidative stress and mitochondrial apoptosis in hepatic and cardiac tissues. The schematic is based on the present experimental findings and supporting literature; the depicted causal interactions were not directly validated in the current study. Figure generated using BioRender Alam-ElDein, K. (2026) https://BioRender.com/0alucsm.

One important detail of the cisplatin-induced injury that is emphasized in the given research paper is the breakdown of endogenous antioxidant systems [28]. Enzymatic and non-enzymatic antioxidant markers such as GSH, GPx, GR, SOD and CAT were all considerably depleted in hepatic and cardiac tissues [29]. On a trace element level, selenium plays a vital role in the structural or catalytic functions of a number of selenoproteins involved in the maintenance of oxidative balance [6,11,30,31]. Glutathione peroxidases and thioredoxin reductases, specifically, require the incorporation of selenium at the active sites to detoxify hydrogen peroxide and lipid hydroperoxides [32]. This sharp loss in GPx activity after cisplatin exposure is perhaps not only the result of oxidative exhaustion but also the result of the decreased capacity of selenium-dependent enzymes [33], as shown in Figure 14.

Suppression of the expression of Nrf2 concomitantly supports the high level of disturbance in intrinsic antioxidant signaling [34]. Nrf2 coordinates the transcription of antioxidant enzyme and phase II detoxification protein encoding genes [19,34]. In physiological states, Nrf2 is suppressed by KEAP-1-mediated degradation by the proteasomes, but oxidative signals typically trigger Nrf2 stabilization and nucleus translocation [35]. Nevertheless, prolonged and excessive ROS generation seems to exceed this adaptive mechanism, leading to the depletion of Nrf2 action and gradual loss of antioxidants [36]. The KEAP-1 upregulation seen in cisplatin-treated animals indicates a strengthening of the Nrf2 degradation pathways, thus enhancing an oxidative vulnerability [37].

The administration of RUT-SeNPs was able to recover the Nrf2 expression and decrease the levels of KEAP-1 in liver and heart tissues. This was accompanied by redox responsive transcription reactivation and high recovery of GSH contents and antioxidant enzyme activities [38]. The increased redox resilience is probably due to the increased selenium bioavailability of the nanoparticulate formulation [39,40]. Selenium nanoparticles have controlled reactivity and reduced toxicity compared to inorganic sodium selenite which shows narrow safety margins and introduces pro-oxidant activity in some circumstances. Their small size allows them to be taken up by the cell and delivered to the intracellular space with constant amounts of selenium, which aid in the efficient production and functioning of selenoproteins [41].

Another layer of redox modulation is the rutin capping component. Rutin has several phenolic hydroxyl groups that can neutralize free radicals via hydrogen donation [42]. In addition to direct scavenging, flavonoids have been reported to mediate redox-sensitive signaling pathways such as the activation of Nrf2 [43]. When selenium and rutin are incorporated into a nanoparticle platform, their synergetic cytoprotective effects are likely to be observed: selenium improves the enzymatic detoxification process, and rutin suppresses the growth of radicals and stabilizes the surfaces of nanoparticles [17]. The noted physicochemical stability, characterized by the nanoscale size distribution and desirable surface charge, might further optimize biodistribution and tissue penetration [44].

Inflammation is the second, but not less significant factor in cisplatin causing tissue damage [45]. High concentrations of TNF-α, IL-6, NF-kB, COX-2, TLR4 and MAPK suggest the stimulation of pro-inflammatory signaling pathways in reaction to oxidative stress [20]. TLR4 is an upstream sentinel receptor that perceives damage related molecular signals produced in the event of cellular injury [46,47]. TLR4 stimulation activates MyD88-dependent pathways, which eventually lead to the phosphorylation of MAPK and nuclear translocation of NF-kB [48]. NF-kB once activated, increases transcription of pro-inflammatory cytokines and enzymes like COX-2, further continuing the process of inflammatory amplification and facilitating further tissue damage [49].

Close association with immune and inflammatory regulation has been associated with selenium status [5]. Sufficiency in selenium facilitates normal cytokine synthesis and restrains hyperactive NF-kB expression via redox-sensitive pathways [50]. In the current work, RUT-SeNPs treatment significantly reduced the expression of TLR4 and MAPK, and the levels of NF-kB activation were also reduced, resulting in a decrease in the concentrations of downstream cytokines [51]. These results imply that selenium nanoparticles do not only strengthen antioxidant defenses but also reduce the inflammatory cascades of signal transduction, which is reinforced by oxidative stress [52]. RUT-SeNPs prevent the oxidative damage-to-inflammation feed-forward loop by stabilizing intracellular thiol redox states and inhibiting the activation of transcription factors by ROS [32].

**Figure 15:**
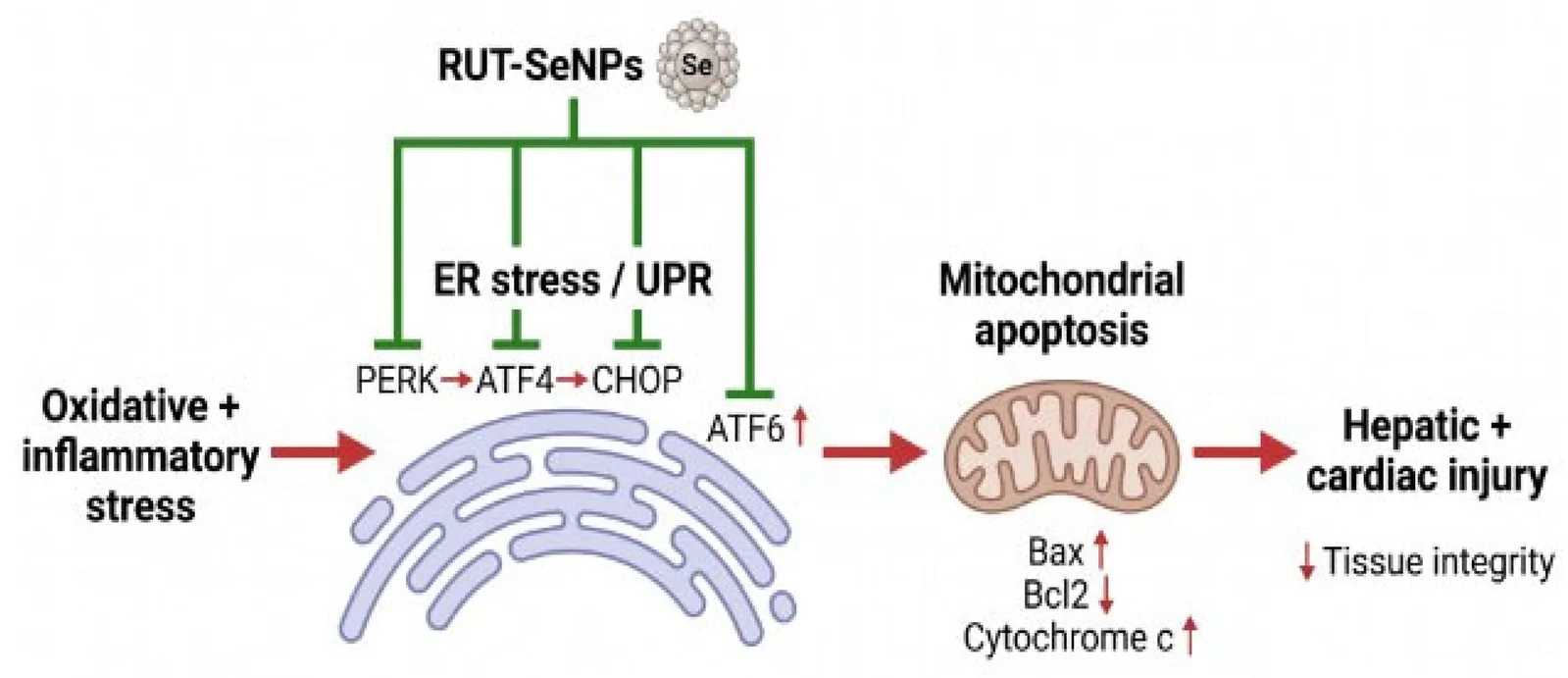
Proposed mechanistic model linking RUT-SeNP treatment with modulation of ER stress-related gene expression and mitochondrial apoptotic markers in cisplatin-induced hepatic and cardiac injury. The schematic is based on the present findings and supporting literature and represents a hypothetical mechanistic interpretation rather than a directly validated pathway. Figure generated using BioRender Alam-ElDein, K. (2026) https://BioRender.com/32m3lpg.

A major mechanistic extension of the present study is the demonstration that cisplatin-induced hepato-cardiac toxicity is accompanied by the activation of ER stress signaling. Cisplatin significantly increased the mRNA expression of *PERK*, *ATF4*, *ATF6*, and *CHOP* in both liver and heart tissues, indicating activation of the unfolded protein response (UPR). The ER is highly sensitive to oxidative imbalance because correct protein folding depends on a tightly controlled redox environment [53]. Excessive ROS production, depletion of antioxidant defenses, and sustained inflammatory signaling can disturb ER proteostasis, resulting in the accumulation of misfolded proteins and activation of ER-resident stress sensors [54]. Among these, PERK activation initiates downstream ATF4 signaling, which can promote adaptive responses during transient stress but stimulates CHOP-mediated apoptosis when ER stress becomes persistent or excessive [55]. The concurrent increase in *ATF6* expression further indicates broad activation of UPR-associated signaling rather than an isolated alteration in a single ER-stress branch [56]. Thus, the present result suggest that ER dysfunction constitutes an intermediate mechanistic link connecting cisplatin-induced oxidative/inflammatory stress with the activation of cell-death pathways. This interpretation is supported by recent evidence showing that cisplatin-induced hepatic injury involves activation of the PERK/ATF4/CHOP axis and ER stress-associated apoptosis as proposed in Figure 15.

Notably, RUT-SeNPs produced the most pronounced suppression of cisplatin-induced *PERK*, *ATF4*, *ATF6*, and *CHOP* upregulation in both hepatic and cardiac tissues. This observation suggests that the protective effect of RUT-SeNPs extends beyond direct antioxidant and anti-inflammatory activities to include the preservation of intracellular protein-folding homeostasis. By reducing ROS accumulation through rutin-associated radical scavenging and selenium-dependent antioxidant reinforcement, RUT-SeNPs may reduce the oxidative burden imposed on the ER, thereby restricting the transition from adaptive UPR activation to maladaptive CHOP-driven death signaling [57,58]. The reduction in CHOP expression is particularly important because CHOP promotes apoptosis through the modulation of Bcl-2 family proteins, mitochondrial dysfunction, and increased susceptibility to oxidative injury [59]. Therefore, suppression of the PERK/ATF4/CHOP and ATF6 pathways provides a mechanistically plausible explanation for the subsequent anti-apoptotic effect observed following RUT-SeNPs administration. Previous work has demonstrated that cisplatin-related hepatic injury can be attenuated via therapeutic interventions targeting ER stress and apoptosis, supporting the biological significance of this pathway in the current model [13,60].

The activation of oxidative, inflammatory, and ER-stress pathways ultimately converged on mitochondrial apoptotic signaling [61]. In the present study, cisplatin significantly upregulated the pro-apoptotic gene *Bax*, suppressed the anti-apoptotic gene *Bcl2*, and increased cytochrome c levels in both hepatic and cardiac tissues. This molecular pattern indicates the disruption of mitochondrial membrane integrity and activation of the intrinsic apoptotic pathway [62]. Bax facilitates mitochondrial outer membrane permeabilization, whereas Bcl-2 acts as a major inhibitor of this process. Accordingly, the cisplatin-induced shift toward Bax predominance would favor mitochondrial release of cytochrome c into the cytosol, where it initiates apoptosome formation and downstream execution of apoptotic cell death [63]. The elevation of cytochrome c levels in the present study therefore provides an important molecular bridge between the observed oxidative/ER stress disturbances and the progression of hepatic and myocardial cellular loss. Previous investigations have similarly demonstrated involvement of the Bax/cytochrome c/caspase-3 apoptotic axis in cisplatin-induced hepatic injury [8].

RUT-SeNPs markedly restored apoptotic homeostasis by reducing *Bax* expression, recovering *Bcl2* expression, and decreasing cytochrome c levels toward those of the control group. These changes indicate that RUT-SeNPs preserve mitochondrial membrane stability and restrict the initiation of mitochondria-dependent apoptosis. Their anti-apoptotic efficacy is likely secondary to coordinated modulation of upstream damaging signals: restoration of KEAP-1/Nrf2-controlled antioxidant defense limits oxidative injury; inhibition of TLR4/MAPK/NF-κB signaling reduces inflammatory amplification; and attenuation of PERK/ATF4/CHOP and ATF6 activation decreases ER stress-associated apoptotic commitment [64]. Therefore, mitochondrial protection by RUT-SeNPs should not be interpreted as an isolated endpoint, but rather as the downstream consequence of broad protection against the interconnected molecular drivers of cisplatin toxicity.

The additional injury was further exacerbated due to apoptotic imbalance when cisplatin was administered. The up regulation of Bax and down regulation of Bcl-2, which are observed, indicates the activation of the intrinsic mitochondrial apoptotic route [65]. Mitochondrial membrane permeabilization, which facilitates the release of cytochrome c and the caspase cascade, is encouraged by oxidative stress and inflammatory mediators [66]. Antioxidant enzymes that require selenium play a vital role in ensuring the redox balance in the mitochondrion as well as mitigating the formation of an apoptotic signal [67]. The regaining of Bax/Bcl-2 equilibrium after RUT-SeNPs treatment implies the maintenance of mitochondrial integrity and inhibition of programmed cell death [68]. The coordinated enhancement of the redox condition, anti-inflammatory effect, and apoptotic control reflects the interdependence of these pathologic events [69].

Even though sodium selenite and rutin separately exhibited protective effects that are measurable, RUT-SeNPs were found to be much more effective in terms of normalizing biochemical, oxidative, inflammatory, and apoptotic parameters [18]. This amplified activity presumably indicates augmented pharmacodynamics and pharmacokinetics of the nanoparticulate selenium, such as prolonged release, augmented cellular uptake, and shortened systemic toxicity [70].

Collectively, the present findings indicate that cisplatin induces hepato-cardiac injury through an interconnected cascade involving oxidative stress, inflammation, ER stress, and mitochondrial apoptosis. Cisplatin increased lipid peroxidation and disrupted KEAP-1/Nrf2-regulated antioxidant defense, with the depletion of GSH and suppression of GR, GPx, SOD, and CAT activities. This redox disturbance was accompanied by the activation of TLR4/MAPK/NF-κB inflammatory signaling and elevation of TNF-α, IL-6, and COX-2 levels. In parallel, cisplatin activated ER-stress signaling through increased PERK, ATF4, ATF6, and CHOP expression, which was associated with mitochondrial apoptotic progression, as indicated by Bax upregulation, Bcl2 suppression, and increased cytochrome c levels. RUT-SeNPs effectively counteracted these alterations by restoring antioxidant signaling, suppressing inflammatory and ER-stress pathways, and preserving mitochondrial apoptotic balance, thereby explaining their superior hepato-cardioprotective effect compared with rutin or sodium selenite alone.

Although the present findings demonstrate broad molecular protection by RUT-SeNPs, further studies are required to confirm protein-level activation of ER-stress and apoptotic pathways, particularly phosphorylated PERK, phosphorylated eIF2α, ATF4, ATF6, CHOP, and cleaved caspase-3. Evaluation of tissue selenium distribution, selenoprotein activity, long-term safety, and possible interference with the antitumor efficacy of cisplatin would also strengthen the translational relevance of this nanoformulation.

A principal limitation of the present study is that ER stress-related endpoints were evaluated exclusively at the transcriptional level through measurement of PERK, ATF4, ATF6, and CHOP mRNA expression. Protein abundance, phosphorylation-dependent activation, ATF6 cleavage, and functional indices of the unfolded protein response were not assessed. Additional Western blot analyses could not be performed because no residual hepatic or cardiac tissue samples remained available after completion of the planned biochemical, molecular, and histological analyses. Consequently, the present findings demonstrate the modulation of ER stress-related gene expression but do not directly confirm the activation or attenuation of ER stress. Future studies should include protein-level assessment of phosphorylated PERK, phosphorylated eIF2α, ATF4, cleaved ATF6, GRP78, and CHOP, together with appropriate functional evaluation of ER proteostasis.

## 4. Materials and Methods

### 4.1. Materials

Analytical-grade cisplatin (CAS No. 15663-27-1), sodium selenite (CAS No. 10102-18-8), and rutin (CAS No. 207671-50-9) were purchased from Sigma Chemical Co. (St. Louis, MO, USA).

### 4.2. Preparation of RUT-SeNPs

**Figure 16:**
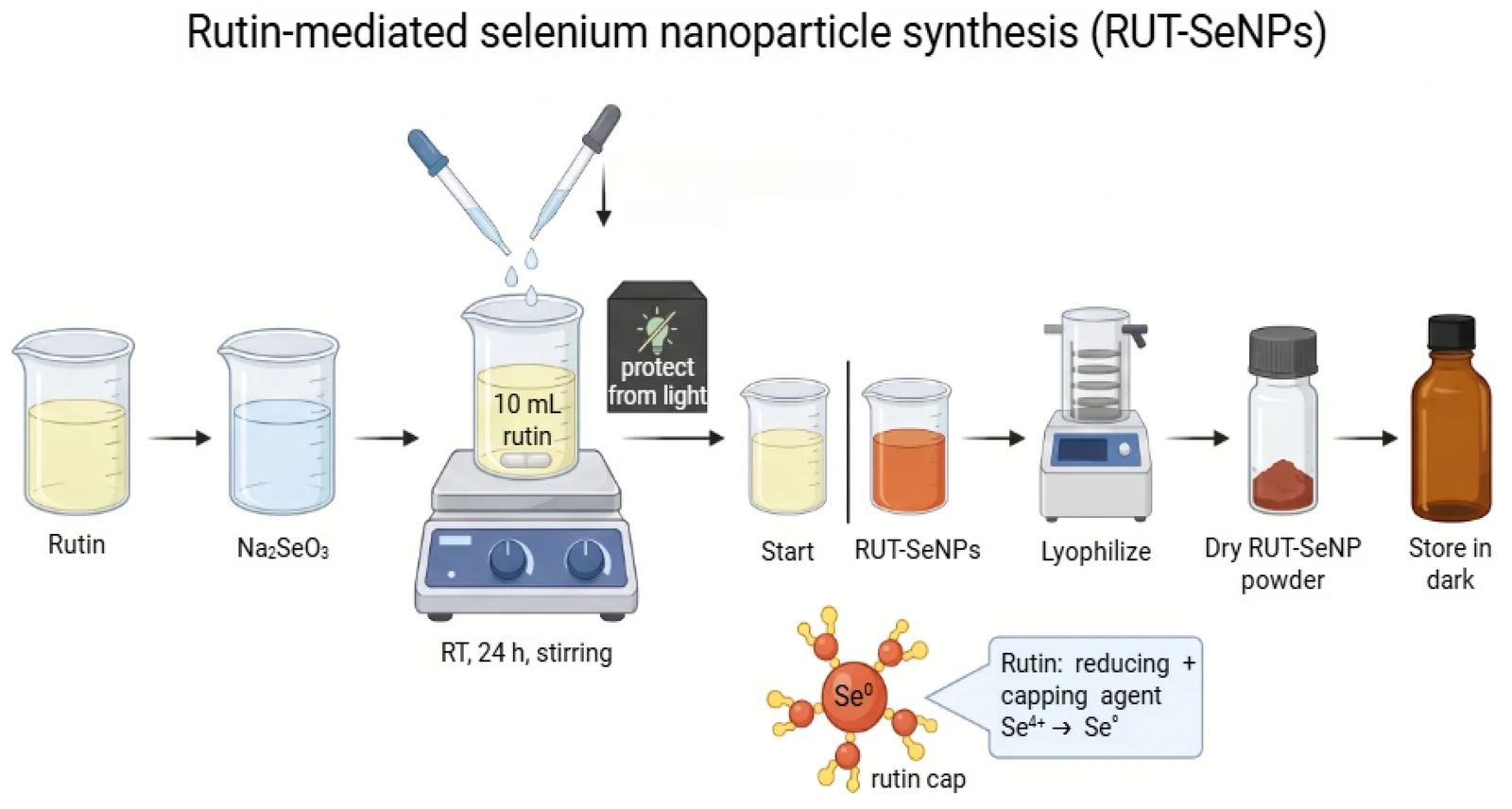
Schematic illustration of the green synthesis of rutin-mediated selenium nanoparticles. Figure generated using biorender, Alam-ElDein, K. (2026) https://BioRender.com/wb47zny.

The selenium nanoparticles (RUT-SeNPs) were prepared using a reduction process that is environmentally friendly, with rutin acteing as a reducing and stabilizing agent. Briefly, 10 mL of an aqueous solution of sodium selenite (10 mM) was added progressively to 10 mL of rutin solution (5 mg/mL) with constant magnetic stirring. To control the formation and growth of the nanoparticles, the reaction system was held at ambient temperature for 24 h to ensure complete reduction of Se^4+^ ions. The formation of elemental selenium nanoparticles was first demonstrated by a change in the color of the reaction mixture (Figure 16).

In an attempts to maintain redox stability and to avoid the reoxidation of selenium species by light, all preparative procedures were performed under light-protected conditions. After completion of the reaction, the resulting colloidal dispersion was lyophilized in a vacuum freeze-drying apparatus (Labconco FreeZone 4.5 L, Marshall Scientific, Hampton, NH, USA) to produce a stable dry formulation of nanoparticles. The obtained RUT-SeNPs powder was collected and stored in the dark.

### 4.3. Physicochemical Characterization

Dynamic light scattering (DLS) and electrophoretic light scattering to measure the zeta potential were used to determine the hydrodynamic diameter, polydispersity index (PDI), and surface charge of the synthesized RUT-SeNPs. These studies were performed at the Nano Center, Faculty of Engineering, Capital University, Cairo, Egypt, in order to measure the particle size distribution, colloidal homogeneity and stability of the dispersion under controlled experimental conditions.

The structural morphology and nanoscale structural peculiarities of RUT-SeNPs were also studied using transmission electron microscopy (TEM). The measurements and sample preparation were conducted at the Nano Center of the Faculty of Science of Al-Azhar University, Cairo Egypt, which allowed measuring the geometry of the particles, their uniformity in size, and in their aggregation behavior.

The crystalline structure of the rutin-mediated selenium nanoparticles (RUT-SeNPs) was characterized via X-ray diffraction (XRD) analysis at the National Research Center (NRC), Giza, Egypt. The dried nanoparticle powder was examined using radiation over an appropriate 2θ scanning range. The resulting diffraction pattern was analyzed to confirm the crystalline nature and formation of elemental selenium nanoparticles.

To verify the synthesis of the nanoparticles and describe their optical characteristics, ultraviolet–visible (UV–Vis) spectrophotometric analysis was used. The absorption spectra of selenium, rutin and RUT-SeNPs were measured at the Nano Center, Faculty of Engineering, Capital University, Cairo, Egypt, in order to identify typical spectral shifts that could demonstrate the formation of selenium nanoparticle and functionalization of the surfaces.

### 4.4. Experimental Design

Thirty-five male Wistar albino rats, each weighing between 200 and 220 g, were obtained from the Animal House at the Faculty of Science, Helwan University, Cairo, Egypt. The rats were maintained under standard laboratory conditions, with a temperature of 22 ± 2 °C and a 12-h light/dark cycle, and were given free access to a standard pellet diet and water. After a one-week acclimatization period, the rats were randomly divided into five groups, with seven rats per group. All experimental procedures were conducted in accordance with the guidelines of the Ethics Committee of School of Biotechnology, Badr University in Cairo, Egypt (protocol code BUC-IACUC/BIOT/141/A/2025—approved on 14 February 2026).

Animals were randomly allocated into five experimental groups, each comprising seven rats, to evaluate the protective effects of the tested compounds against cisplatin (CIS)-induced cardiotoxicity.

Group I (CON): Rats received normal saline (0.9% NaCl) orally throughout the experimental period and intraperitoneal (ip.) saline injections on the corresponding day of cisplatin administration, serving as a negative control.

Group II (CIS): Rats in this group were administered cisplatin intraperitoneally with single dose of 7.5 mg/kg body weight at day 20, following the established protocol [71,72].

Group III (CIS + Rutin): Rats in this group received rutin orally at dose of 100 mg/kg per day for 21 consecutive days [71]. Then rats of this group were injected with single dose of cisplatin 7.5 mg/kg, ip at day 20 [71,72].

Group IV (CIS + Selenium): Rats in this group were treated orally with sodium selenite as precursor for selenium (Na_2_SeO_3_) at a dose of 0.5 mg/kg body weight per day for 21 consecutive days [73]. Then, rats in this group were injected with single dose of cisplatin 7.5 mg/kg, ip. at day 20 [71].

Group V (CIS + RUT-SeNPs): Rats in this group treated orally with selenium nanoparticles biosynthesized using rutin (RUT-SeNPs) at a dose of 0.5 mg/kg body weight per day for 21 consecutive days [5,74], Then rats in this group were injected with single dose of cisplatin 7.5 mg/kg, ip. at day 20 [71].

The sample size (*n* = 7) was determined based on previous studies using comparable experimental models and treatment regimens, taking into account ethical considerations to minimize animal use while ensuring adequate statistical comparisons among groups [5,10].

Within 24 h after the final treatment, the animals were euthanized under deep anesthesia induced by intraperitoneal ketamine/xylazine (100 and 10 mg/kg body weight, respectively). Blood was collected via cardiac puncture, allowed to clot at 37 °C for 20–30 min, centrifuged at 3000 rpm for 15 min, and the obtained serum was aliquoted and stored at −20 °C for biochemical analyses. Liver and heart tissues were rapidly excised, rinsed with ice-cold 10 mM PBS, and divided for further investigations: portions were fixed in neutral buffered formalin for histopathological examination; other samples were homogenized in ice-cold PBS, centrifuged at 3600 rpm for 10–15 min, and the supernatants were preserved at −80 °C for oxidative stress and biochemical assessments; while separate tissue fractions were homogenized in TRIzol^®^ reagent under RNase-free conditions for total RNA extraction and subsequent gene expression analysis.

### 4.5. Assessment of Hepatic Function

Serum hepatic functional indicators were measured according to the instructions of the manufacturers using commercially available assay kits. ALT and AST levels were measured using commercial kits from BioVision (Cat. Nos. E4325-100 and E4321-100, respectively; Milpitas, CA, USA). The levels of total bilirubin and albumin in the samples were estimated using colorimetric assay kits from Elabscience (Cat. Nos. K553 and K554, respectively). The collected data were measured twice, and absorbance was determined at the given wavelengths using a calibrated microplate reader.

### 4.6. Assessment of Cardiac Function

Serum cardiac injury and functional biomarkers were identified using rat-specific immunoassay kits according to the protocols given by the respective manufacturers. The levels of CK-MB, cTnT, and ANP were determined using ELISA kits from Elabscience (Cat. Nos. E-CL-R0722 and E-EL-R1253, E-EL-R0017, respectively), while the levels of FABP3 and MYL3 were determined using Proteintech kits (Cat. Nos. 60280-1-Ig, and 10913-1-AP, respectively).

### 4.7. Gene Expression Analysis

Total RNA was extracted from cardiac and hepatic tissues using TRIzol™ reagent, followed by cDNA synthesis using the MultiScribe™ Reverse Transcriptase Kit. RT-qPCR was performed using Power SYBR™ Green Master Mix on an Applied Biosystems 7500 System to evaluate the mRNA expression of antioxidant response markers (*Nrf2* and *KEAP-1*), apoptotic markers (Bax and Bcl-2), and ER stress markers (*CHOP*, *ATF4*, *ATF6*, and *PERK*). The primer sequences are listed in Table 1. Gene expression was normalized to that of β-actin, and relative expression was determined using the 2^−ΔΔCt^ method.

### 4.8. Lipid Peroxidation Assessment

Lipid peroxidation and redox regulating signals were assessed through the determination of malondialdehyde (MDA). The concentration of MDA was measured as an indicator of oxidative damage to cell membranes using the Lipid Peroxidation (MDA) Assay Kit (Cat. No. K739-100, BioVision, Milpitas, CA, USA).

### 4.9. Assessment of Antioxidant Defense System

Antioxidant status was defined by the measurement of reduced glutathione (GSH) content and the activity of main antioxidant enzymes, such as glutathione reductase (GR; Cat. No. KE00840, Proteintech, Rosemont, IL, USA), glutathione peroxidase (GPx; Cat. No. K762-100, BioVision), superoxide dismutase (SOD; Cat. No. K335-100, BioVision) and catalase (CAT; Cat. No. K773-100, BioVision).

### 4.10. Assessment of Inflammatory Markers

Hepatic and cardiac inflammatory responses were assessed by measuring TNF-α, IL-6, NF-κB, and COX-2 levels using rat-specific immunoassay kits. TNF-α and IL-6 were quantified using ELISA kits from Cloud-Clone Corp (Cat. Nos. SEA133Ra and SEA079Ra, respectively, Katy, TX, USA), NF-κB was determined using a FineTest ELISA kit (Cat. No. ER1186, FineTest, Boulder, CO, USA), and COX-2 expression was analyzed using a Proteintech antibody-based assay (Cat. No. 27308-1-AP).

### 4.11. Assessment of Cytochrome C, MAPK and TLR4 Signaling

Cardiac and hepatic cytochrome c was assessed using a commercial ELISA kit from mybiosource (Cat No. MBS9304546), while MAPK and TLR4 levels were detected using Proteintech immunoassay kits (MAPK, Cat. No. PK30019; TLR4, Cat. No. 19811-1-AP) following the manufacturer’s instructions.

### 4.12. Immunohistochemical Analysis

To compare the expression of NF-kB (1:100 dilution, Cat. No. ab32536, Abcam, Cambridge, UK) in cardiac and hepatic tissues, immunohistochemical staining was conducted. The tissue sections on paraffin were deparaffinized, rehydrated, and incubated with antigen retrieval based on a suitable buffer system (4–5 µm paraffin-embedded tissue sections). Primary antibodies against NF-kB were incubated under optimal conditions after blocking endogenous peroxidase activity. After washing, sections were incubated with a suitable secondary antibody conjugated to horseradish peroxidase (HRP), and immunoreactivity was visualized using a chromogenic substrate such as 3,3′-diaminobenzidine (DAB). The sections were counterstained with hematoxylin, mounted and observed under a light microscope to determine the localization/relative expression of NF-kB in cardiac and liver tissues [75,76,77]. A semi-quantitative immunohistochemical method was used to determine the localization and relative intensity of the expression of target proteins in the tissue sections. The stained sections were examined using ImageJ Fiji software (version 1.54n), which was used to perform color deconvolution and subsequent quantitative analysis of the image. This protocol allowed a quantitative evaluation of the immunoreactive areas percentage of 5 non-overlapping fields and offered replication measurements of protein expression among the experiment groups [78,79].

### 4.13. Histopathological Evaluation (Hematoxylin and Eosin Staining)

Excised cardiac and hepatic tissues were immediately fixed in 10% neutral buffered formalin to preserve morphological integrity, followed by routine processing involving graded alcohol dehydration, xylene clearing, and paraffin embedding. Paraffin blocks were sectioned at 4–5 µm thickness using a rotary microtome, and the obtained sections were mounted on clean glass slides. The tissue sections were stained with hematoxylin and eosin (H&E) according to established histological procedures. Microscopic examination was performed using a light microscope to assess architectural integrity and pathological alterations within myocardial and hepatic tissues. Histopathological evaluation was performed by an experienced pathologist who was blinded to the experimental group allocation and treatment identity during microscopic examination and scoring [80,81].

### 4.14. Statistical Analysis:

Data normality was assessed using the Shapiro–Wilk test. Normally distributed data were expressed as the mean ± standard deviation (SD) and analyzed using one-way analysis of variance (ANOVA) followed by Tukey’s post hoc test for multiple comparisons. The Statistical Package from Graphpad Prism 9.0 was used to compare the treated groups. Changes between means were considered significant when *p* < 0.05. The percentage difference, representing the variation with respect to the control group, was calculated using following equation:

$$\%\;\mathrm{of}\;\mathrm{Difference}\;=\;\frac{\mathrm{Treat}ed\;\mathrm{value}\;–\;\mathrm{control}\;\mathrm{value}}{\mathrm{Control}\;\mathrm{value}}\times 100$$

## 5. Conclusions

In conclusion, RUT-SeNPs provided substantial protection against cisplatin-induced hepatic and cardiac injury. This protection was accompanied by the restoration of redox balance, suppression of inflammatory mediators, reduced mRNA expression of the ER stress-related genes PERK, ATF4, ATF6, and CHOP, and modulation of mitochondrial apoptosis-associated markers. Therefore, the findings support an association between RUT-SeNP treatment and reduced ER stress-related transcriptional responses but do not directly demonstrate the attenuation of ER stress at the protein or functional level.

## Figures and Tables

**Table 1:** Primer Sequences Used for qRT-PCR Analysis.

Marker	Forward Sequence	Reverse Sequence
*Nrf2*	GCCAGCTGAACTCCTTAGAC	GATTCGTGCACAGCAGCA
*KEAP-1*	TGAAGTGACCCGCTTGACAT	CCCAGGAGAAGTGTCACAGAA
*Bax*	ACACCTGAGCTGACCTTGGA	AGTTCATCGCCAATTCGCCT
*Bcl-2*	CTGGTGGACAACATCGCTCT	GCATGCTGGGGCCATATAGT
*CHOP*	AATTGGGGGCACCTATATCTCATC	GGCTTTGGGAGGTGCTTGT
*ATF4*	TGGCTATGGATGGGTTGGTC	GCTCATCTGGCATGGTTTCC
*ATF6*	CCAGAGGCTCAAAGTCCCAAGT	TGACATGGAGGTGGAGGGATAT
*PERK*	GATACGGCATTTGGCTTGGG	CCCATGATTCTCGGCATCCA

## Data Availability

The original contributions presented in this study are included in the article. Further inquiries can be directed to the corresponding authors.

## Abbreviations

The following abbreviations are used in this manuscript:

Abbreviation	Full Term
ALB	Albumin
ALT	Alanine aminotransferase
ANOVA	Analysis of variance
ANP	Atrial natriuretic peptide
AST	Aspartate aminotransferase
ATF4	Activating transcription factor 4
ATF6	Activating transcription factor 6
Bax	Bcl-2-associated X protein
Bcl-2	B-cell lymphoma 2
CAT	Catalase
cDNA	Complementary DNA
CHOP	C/EBP homologous protein
CIS	Cisplatin
CK-MB	Creatine kinase-MB
COX-2	Cyclooxygenase-2
cTnT	Cardiac troponin T
DAB	3,3′-Diaminobenzidine
DLS	Dynamic light scattering
ELISA	Enzyme-linked immunosorbent assay
ER	Endoplasmic reticulum
FABP3	Fatty acid-binding protein 3
GPx	Glutathione peroxidase
GR	Glutathione reductase
GSH	Reduced glutathione
H&E	Hematoxylin and eosin
IACUC	Institutional Animal Care and Use Committee
IHC	Immunohistochemistry
IL-6	Interleukin-6
i.p.	Intraperitoneal
KEAP-1	Kelch-like ECH-associated protein 1
MAPK	Mitogen-activated protein kinase
MDA	Malondialdehyde
mRNA	Messenger RNA
MYL3	Myosin light chain 3
Na_2_SeO_3_	Sodium selenite
NF-κB	Nuclear factor kappa B
Nrf2	Nuclear factor erythroid 2-related factor 2
PBS	Phosphate-buffered saline
PDI	Polydispersity index
PERK	Protein kinase RNA-like endoplasmic reticulum kinase
qRT-PCR	Quantitative reverse transcription polymerase chain reaction
ROS	Reactive oxygen species
RUT	Rutin
RUT-SeNPs	Rutin-mediated selenium nanoparticles
SeNPs	Selenium nanoparticles
SEM	Standard error of the mean
SOD	Superoxide dismutase
TEM	Transmission electron microscopy
TLR4	Toll-like receptor 4
TNF-α	Tumor necrosis factor-alpha
TRIzol	Total RNA isolation reagent
UPR	Unfolded protein response
UV–Vis	Ultraviolet–visible spectroscopy
XRD	X-ray diffraction
